# A New Current Fingerprinting Sensing Method for Adaptive Protection of PV-Integrated Distribution Networks

**DOI:** 10.3390/s26196323

**Published:** 2026-10-07

**Authors:** Ahmed A. Salem, Emad Eldeen Omran, Azza A. ElDesouky, Hany S. E. Mansour, Mohamed A. A. Mohamed, Eyad S. Oda

**Affiliations:** 1 Department of Electrical Engineering, Faculty of Engineering, Suez Canal University, Ismailia 41522, Egypt; ahmed_salem@eng.suez.edu.eg (A.A.S.); hany_salem@eng.suez.edu.eg (H.S.E.M.); eng.eyad.s.s.oda@gmail.com (E.S.O.); 2 Suez Canal Authority, Ismailia 41522, Egypt; emad.eldin.omran@eng.suez.edu.eg; 3 Department of Electrical Engineering, Faculty of Engineering, Port Said University, Port Fouad 42526, Egypt; aza.ahmed@eng.psu.edu.eg; 4 Green Energy Program, Egypt-Japan KOSEN (EJ-KOSEN) Institute of Technology, 10th of Ramadan City 44629, Egypt; 5 WMG (Warwick Manufacturing Group), University of Warwick, Coventry CV4 7AL, UK; 6 Faculty of Engineering, Assiut University, Assiut 71515, Egypt

**Keywords:** fault current sensing, current fingerprinting, operating-mode classification, local positive sequence measurement, instrument transformer accuracy, adaptive protection, grid-following inverter, directional overcurrent relay, linear programming, photovoltaic integration

## Abstract

Grid-following photovoltaic (PV) inverters inject fault currents whose magnitude and phase are set by the control mode active during the disturbance, so directional overcurrent relay (DOCR) settings coordinated for one inverter response can miscoordinate after a mode change. This paper proposes a communication-free adaptive protection scheme built on local sensing. A voltage-adaptive current fingerprinting classifier uses local positive sequence measurements to detect current-limiting (CL), active current priority (ACP) and reactive current priority (RCP) operation, as well as momentary cessation (MC) and asymmetrical faults. The classifier activates the relay setting group prepared offline for the detected mode. With pickup currents fixed, the coordination sub-problem is shown to be a linear program (LP), so every setting group is computed at its exact global optimum. Where the grid code droop law makes ACP and RCP indistinguishable by angle, the classifier declares the ambiguity and activates a group that is feasible for both. Under a class-0.5 instrument transformer noise model, the group placed in service is coordination-valid in 99.8% of cases and the expected residual miscoordination is $3.0\times 10^{-5}$ s, 0.01% of the 0.30 s margin. On the CIGRE medium-voltage benchmark, the scheme satisfies the margin in all operating modes for three-phase, line-to-line (LL) and single-line-to-ground (SLG) faults. The same fault types are then verified at three fault locations on a practical Egyptian 11 kV feeder model, where the sensing layer is confirmed on an electromagnetic transient (EMT) model and the scheme removes the violations of single-setting coordination using an estimated 4 kB of relay memory.

## 1. Introduction

### 1.1. Motivation and Literature Review

The global transition toward low-carbon power systems has accelerated the large-scale deployment of utility-scale photovoltaic (PV) generation at the medium-voltage (MV) distribution level [1,2]. However, this shift has complicated the coordination of overcurrent protection. Unlike conventional synchronous generators, whose fault contribution is governed by well-characterized sub-transient reactance and decays predictably following voltage collapse, a grid-following (GFL) inverter regulates its output current through high-bandwidth inner control loops and enforces a programmable current limitation strategy during voltage disturbances. This departure from classical fault physics undermines the assumptions underpinning conventional directional overcurrent relay (DOCR) coordination and motivates a re-examination of both the problem formulation and the methods used to solve it. The observation underpinning this work is that the active inverter mode is imprinted on the locally measured fault current, so that mode can be sensed and identified at each relay from local positive sequence current and voltage measurements alone. This paper therefore reframes adaptive coordination as a local sensing-and-classification task and develops a strategy-aware protection concept spanning real-time operating-mode sensing, offline setting group optimization, and communication-free online relay operation.

The integration of inverter-based resources (IBRs) into MV distribution networks has been documented extensively as a cause of degraded overcurrent protection selectivity [3,4,5,6]. At the physical level, a GFL inverter operating through a cascaded control structure with current-control bandwidths exceeding 1 kHz does not behave as a voltage-behind-impedance source [7,8]. The phase angle of the GFL fault current is shaped by the active limitation strategy [9,10,11], which grid codes including IEEE 1547-2018 [12], IEEE 2800-2022 [13], and VDE-AR-N 4110 [14] formalize into three dominant modes, namely current limiting (CL), active current priority (ACP) and reactive current priority (RCP). A fourth mode, momentary cessation (MC), suspends current injection during deep voltage sags below a configurable threshold [12,15]. Quantitative studies have established that the effective ratio $I_{f}/I_{p}$ (fault current normalized by the relay pickup current) can vary by more than 26% depending solely on the active inverter mode, without any change in network topology [9,11,16]. This quantitative sensitivity to inverter strategy motivates a closer, physics-based comparison of why GFL and grid-forming (GFM) inverters respond so differently to faults, presented in Section 2; the resulting strategy-aware coordination framework is developed in the remainder of this paper.

Metaheuristic optimization has become the dominant computational paradigm for determining optimal time multiplier setting (TMS) vectors in overcurrent protection coordination [17,18,19,20,21,22]. Differential evolution was among the earliest population-based methods applied to DOCR problems [23], followed by biogeography-based optimization (BBO) [20] and hybrid genetic algorithm (GA) approaches [18]. More recent contributions have applied particle swarm optimization (PSO) [19], the whale optimization algorithm (WOA), the grey wolf optimizer (GWO) [21], the war optimization algorithm [24], the walrus optimizer [25], the jellyfish search algorithm [26] and the elite marine predators algorithm [27] to relay coordination benchmarks ranging from IEEE standard test systems to International Electrotechnical Commission (IEC) microgrid configurations. Setting group schemes with non-standard relay curves have also been proposed for overcurrent and ground fault relays [28], and load forecasting has been used to update settings ahead of the operating point [3]. In parallel, voltage-adaptive relay schemes have been proposed to mitigate coordination failures caused by variable distributed generation (DG) infeed, though most treat the inverter contribution as a simplified equivalent current source rather than a strategy-dependent phasor [29]. Schemes targeting islanded microgrids (MGs) have addressed the N-1 (single-contingency, one-element-out) optimization problem and the coexistence of inverter-dominated and synchronous sources [30,31], while dual-characteristic approaches with fault current limiters have been evaluated on the IEEE 39-bus and 8-bus systems under PV uncertainty [32].

Despite this body of work, nearly all published DOCR coordination studies assume a fixed or worst-case fault current contribution from IBRs, and thus ignore the switching among CL, ACP, and RCP modes that occurs in real deployments [17,18,19,20,21,22].

### 1.2. Research Gap

Several investigations have acknowledged this limitation without fully resolving it:

- Voltage-adaptive overcurrent relay (OCR) schemes [29] addressed the magnitude variation caused by IBR infeed but did not distinguish strategies by phase angle.
- Enhanced voltage-phase-margin (EVPM) methods [33] handled MC only partially and lacked a systematic classification framework.
- A centralized protection scheme using an artificial neural network for fault detection and location [34] and an enhanced decentralized machine-learning-based protection scheme [35] achieved data-driven fault-condition awareness, yet the centralized architecture remains incompatible with existing commercial relay hardware and neither explicitly classifies the active GFL inverter control strategy.
- A dynamic setting group framework for overcurrent and ground fault relays [28] changes the relay characteristic with the operating point, but the group is selected from the network state rather than from the inverter control mode.
- A setting group approach for inverter-interfaced distributed generation (IIDG) [36] and an adaptive protection scheme explicitly accounting for MC [15] were the only prior works to address strategy switching directly; however, neither classified the active strategy in real time from local relay measurements, and the scheme in [15] handled asymmetrical faults only partially.

Table 1 maps each reviewed work against the key capabilities required to fully solve the problem.

Two of these studies come closest to the present work: the setting group scheme for inverter-interfaced distributed generation of Ayvaz [36] and the MC-aware adaptive scheme of Omran et al. [15]. Both switch among pre-computed relay settings, but neither identifies the active inverter control mode from local measurements. Reference [36] selects settings by demand period (peak/off-peak) rather than by inverter strategy, whereas [15] addresses MC without an explicit CL/ACP/RCP classifier and handles asymmetrical faults only partially.

Based on the studies reviewed above, no existing DOCR coordination scheme simultaneously classifies the active GFL inverter strategy in real time from local relay measurements, activates a pre-optimized matched setting group, and handles both MC and asymmetrical faults without any communication infrastructure. This threefold gap leaves MV distribution networks with PV integration systematically exposed to protection failures whenever the inverter transitions between operating modes, a scenario that occurs routinely under varying irradiance, grid voltage disturbances, and utility fault conditions.

### 1.3. Contributions of This Paper

The contributions of this paper are as follows.

(1) A local, communication-free sensing mechanism that identifies CL, ACP and RCP, together with MC and asymmetrical faults, from local positive sequence measurements using analytically derived, voltage-dependent thresholds.
(2) A characterization of that mechanism under a class-0.5 instrument transformer noise model, including the voltage band in which ACP and RCP are intrinsically ambiguous, and a four-output decision (CL/ACP/RCP/ambiguous, AMB) that resolves the ambiguity with a setting group feasible for both modes; the figure of merit is the fraction of coordination-valid setting groups placed in service rather than label accuracy.
(3) A proof that, with fixed pickup currents, the strategy-aware coordination sub-problem is a linear program (LP), so each setting group is obtained at its exact global optimum, together with a strategy switching miscoordination index.
(4) An analysis under imperfect classification that propagates the measured decision statistics through the miscoordination index.
(5) Validation on the CIGRE medium-voltage (MV) benchmark with dual PV farms and a practical Egyptian 11 kV feeder model, using the multi-setting group feature of commercial relays [41,42,43], and verification of the sensing layer on an electromagnetic transient (EMT) model of the relay, the inverter controls and the current transformer.

The present work departs from these two studies in three ways. First, it makes the sensing and classification of the inverter mode the core of the scheme and treats it as a measurement problem in terms of accuracy, noise, and latency. Second, it unifies CL, ACP, RCP, MC, and asymmetrical faults (AS) within a single local classifier governed by analytically derived, network-independent voltage thresholds. Third, it quantifies coordination performance under imperfect classification, bridging the gap between an idealized adaptive scheme and one that can be implemented on existing relays. The setting groups are obtained from a linear program; metaheuristic solvers are used only as benchmarks and are not claimed as a contribution of this work.

The remainder of this paper is organized as follows. Section 2 contrasts the GFL and GFM inverter fault-response mechanisms. Section 3 describes the system modeling, protection configuration, and the coupling of the coordination time interval (CTI) to the inverter mode. Section 4 develops the local sensing and fingerprint classifier and extends it to MC and asymmetrical faults. Section 5 formulates the coordination problem. Section 6 presents Case Study I on the CIGRE MV benchmark, including the classifier sensing performance, the imperfect classification robustness analysis, and the EMT verification; Section 7 validates the scheme on the practical Nediba Egyptian 11 kV feeder model as Case Study II; Section 8 discusses the findings, deployment implications, and limitations; and Section 9 concludes the paper.

## 2. Fault Response of GFL and GFM Inverters

**Figure 1:**
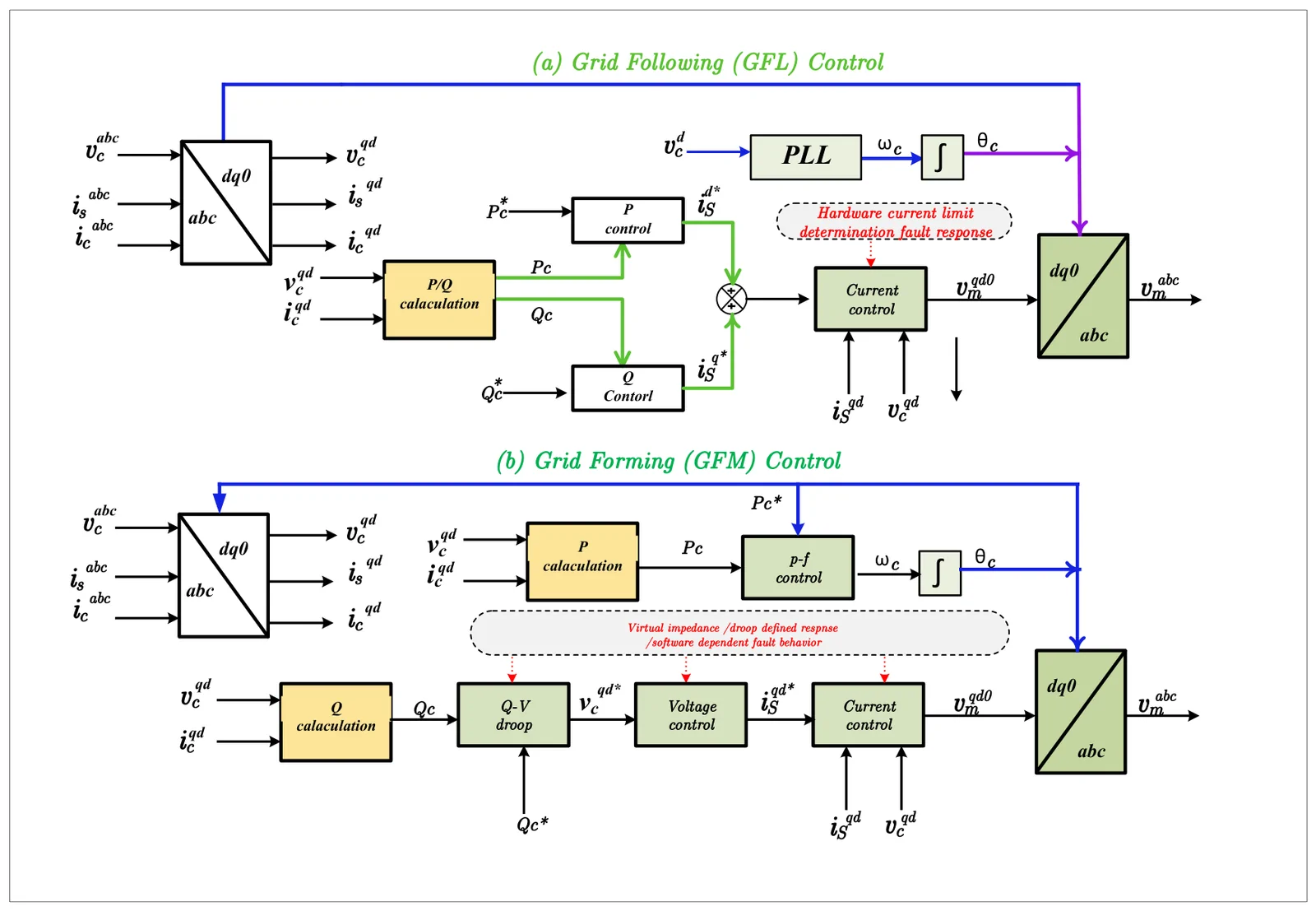
Inverter control structures. (**a**) GFL. (**b**) GFM.

In the GFL scheme of Figure 1a, the converter measures the point-of-common-coupling (PCC) voltage and the converter currents and transforms them into the synchronous dq frame using the angle supplied by a phase-locked loop (PLL), $\theta_{c}\left(t\right)=\int_{0}^{t}\omega_{c}\left(\tau \right)\;d\tau$. Outer P and Q controllers produce the current references $i_{d}^{*}$ and $i_{q}^{*}$, which are hard-limited before the inner current loop,

(1) $$\sqrt{{\left(i_{d}^{*}\right)}^{2}+{\left(i_{q}^{*}\right)}^{2}}\leq I_{\max},$$

where $I_{\max}$ is the converter current limit. Because of limit (1), the inverter behaves as a current source with a ceiling $I_{\max}$ regardless of fault severity. The GFM scheme of Figure 1b replaces the PLL and power controllers by droop laws, $\omega_{c}=\omega_{0}+m_{p}\left(P_{c}^{*}-P_{c}\right)$ and $v_{c}^{d*}=E_{0}+n_{q}\left(Q_{c}^{*}-Q_{c}\right)$, with droop gains $m_{p}$ and $n_{q}$, nominal frequency $\omega_{0}$ and nominal voltage $E_{0}$, so the converter sets its own voltage and frequency. Its fault current is shaped by a software virtual impedance $Z_{v}$ rather than by a hard ceiling [13,44,45].

**Figure 2:**
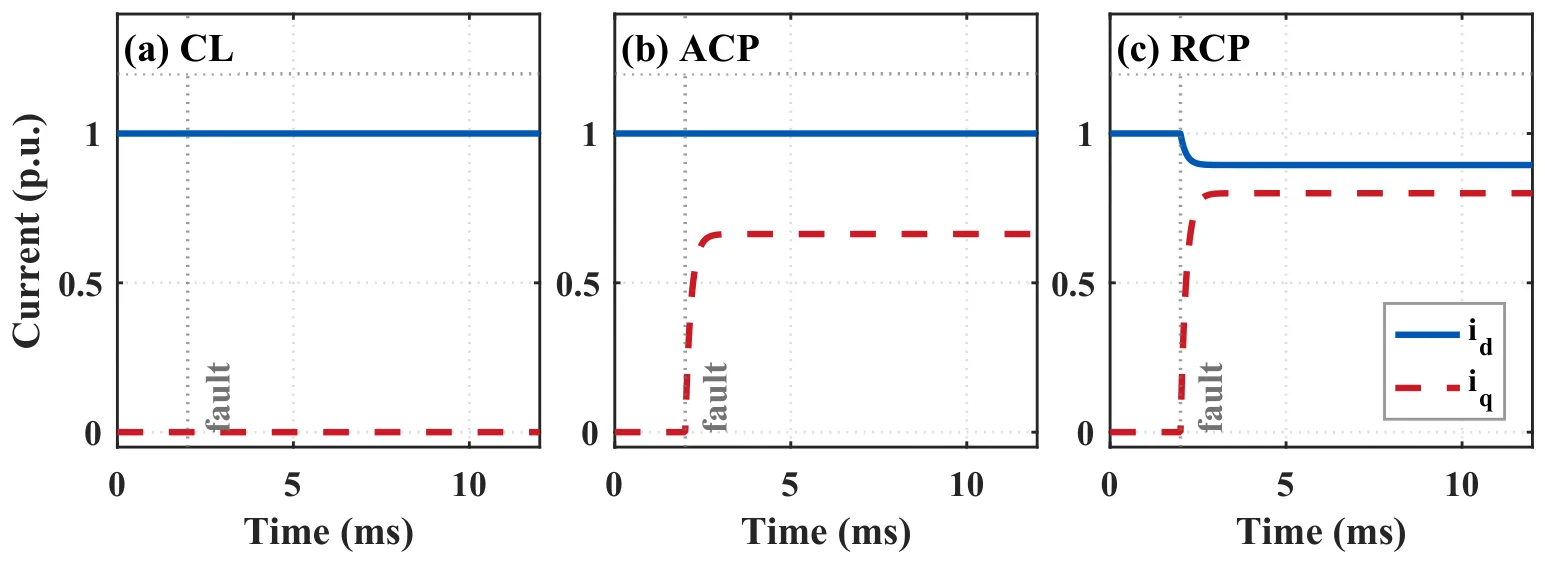
GFL dq-current response to a voltage sag. (**a**) CL. (**b**) ACP. (**c**) RCP.

The GFL inner loop is a PI current controller with a bandwidth above 1 kHz, and the synchronous reference frame (SRF) PLL has a bandwidth of 20–100 Hz [7,8]. The current settles as a first-order response, $\tau_{c}\;di_{d,q}/dt+i_{d,q}=i_{d,q}^{*}$, with time constant $\tau_{c}=1/\left(2\pi f_{c}\right)\approx 0.16$ ms for a loop bandwidth $f_{c}=1$ kHz, so the strategy-dependent steady state is reached within 1–2 ms, well before relay pickup at 10–20 ms (Figure 2), and the active strategy can therefore be identified before a trip decision is taken.

## 3. System Modeling and Protection Configuration

The scheme is evaluated on two networks. The first is the CIGRE medium-voltage benchmark, which carries the classifier and coordination study of Section 4, Section 5 and Section 6 and is described below. The second is a practical Egyptian 11 kV feeder, used in Section 7 to confirm the scheme on a utility network with a recloser–fuse pair. Both were modeled in ETAP and analyzed in MATLAB.

**Figure 3:**
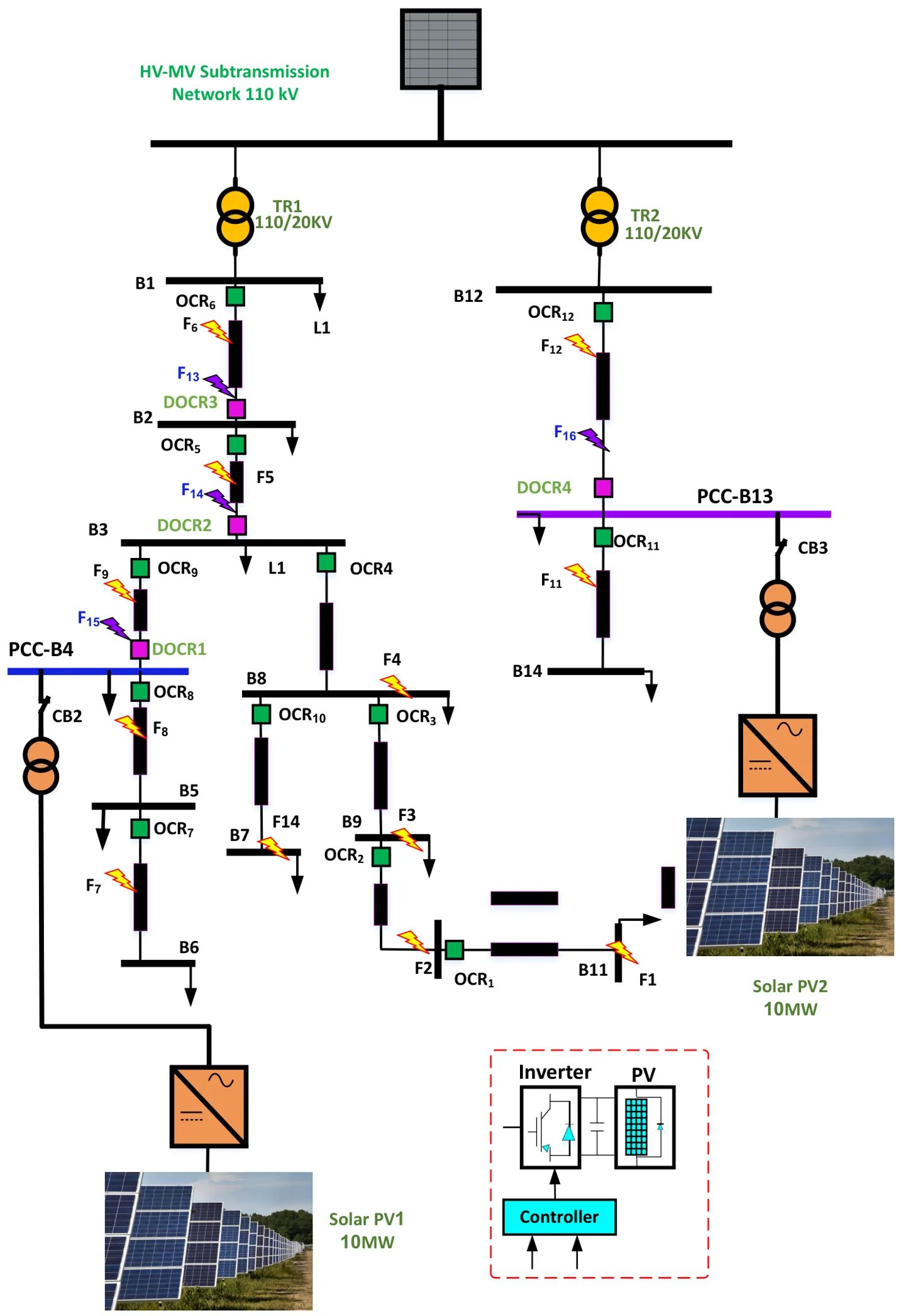
CIGRE 14-bus MV network with two PV farms, relays OCR_1_–OCR_12_ and DOCR_1_–DOCR_4_ (green/magenta markers), and fault points F_1_–F_16_, evaluated for the OCR relays (F_1_–F_12_) and the DOCR pairs (F_13_–F_16_).

**Figure 4:**
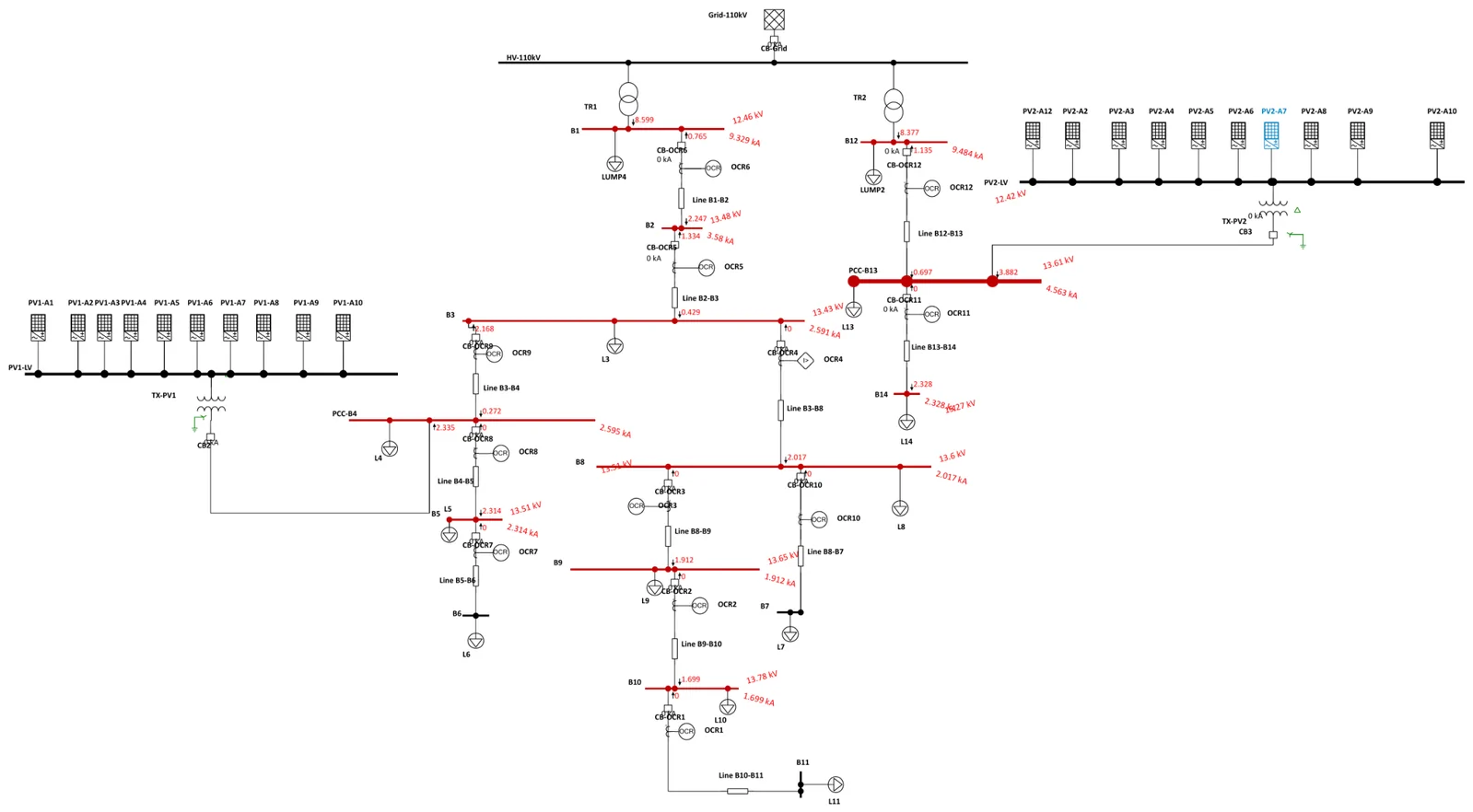
ETAP load-flow model of the network in Figure 3, used to verify the network data; colors and percentages follow ETAP’s default display.

The CIGRE 14-bus MV network, operated at 20 kV, is supplied by two 110/20 kV transformers [46] (Figure 3), with the protection configuration of our earlier study [15]. Two 10 MW PV farms are connected at Buses 4 and 13 through 2/20 kV step-up transformers, each farm comprising ten GFL units with the parameters of Table 2. Fault currents are evaluated in the quasi-steady state reached after inner-loop settling, which justifies the phasor-domain analysis. Under three-phase faults the inverters inject balanced positive sequence current; asymmetrical faults are treated in Section 4.5 and Section 6.4. The short-circuit studies were run in ETAP to IEC 60909 [47] (minimum currents, c=1.0). The relays are IEC 60255-151 [48] standard-inverse elements; their current transformer (CT) ratios and pickup currents are listed in Table 3, and their operating-time characteristic is formulated with the coordination problem in Section 5. The ETAP model is shown in Figure 4 with the bus, line and relay labels used throughout. The sensing layer is further verified on an EMT model of one PV connection, described in Section 6.9.

Five operating modes (OM) are considered. OM1 represents utility-only operation, OM2 represents utility operation with the PV inverter in CL, OM3 represents utility operation with the PV inverter in ACP, OM4 represents utility operation with the PV inverter in RCP, and OM5 represents momentary cessation and/or asymmetrical faults. The inverter operating mode affects the fault current seen by each relay. Therefore, a time multiplier setting vector that satisfies the selectivity margin in one operating mode may not satisfy the same margin in another mode.

## 4. Local Sensing and Voltage-Adaptive Fingerprinting

### 4.1. Measurement Model

The inverter mode is sensed from quantities already available to a numerical feeder relay. The relay estimates positive sequence phasors with a one-cycle recursive estimator and resolves the inverter current into the dq frame [12]. The sensing chain consists of a CT, a voltage transformer (VT), anti-aliasing filtering and phasor estimation; no phasor measurement unit, time synchronization or communication link is required, so the scheme is implementable on existing relay hardware. Because the decision uses the current angle, its reliability is set by the angular accuracy of the instrument transformers. Class-0.5 transformers bound the phase displacement to ±0.9°, i.e., an angular standard deviation σ≤0.8° [49]. Here, the noise is treated as a disturbance to be bounded; in learning-based fault diagnosis it can also be used to improve robustness and generalization [50,51]. Angular error is the main sensing limitation; saturation and PLL effects are assessed separately in Section 6.6 and Section 6.7.

### 4.2. Strategy Fingerprints and Decision Rule

The relay forms two quantities from the measured inverter current: its magnitude $I_{\mathrm{IBR}}$ and the angle fingerprint φ between the current and the measured voltage, the magnitude of which is written $|V_{\mathrm{PCC}}|$ and is expressed in per unit of the nominal voltage,

(2) $$I_{\mathrm{IBR}}=\sqrt{i_{d}^{2}+i_{q}^{2}},\;\varphi =\arctan\left(i_{q}/i_{d}\right),\;i_{d}>0.$$

Throughout this section the inverter carries its rated active current before the fault, that is, a pre-fault loading γ=1 in the notation of Section 4.4. Under CL, rated active current is kept without reactive injection, $i_{d}^{\mathrm{CL}}=I_{r}$, $i_{q}^{\mathrm{CL}}=0$, so $\varphi^{\mathrm{CL}}=0^{\circ }$. Under ACP the headroom above, $I_{r}$ is allocated to reactive support,

(3) $$i_{d}^{\mathrm{ACP}}=I_{r},\;i_{q}^{\mathrm{ACP}}=\sqrt{I_{\max}^{2}-I_{r}^{2}}\approx 0.663\;I_{r},\;\varphi^{\mathrm{ACP}}\approx 33.6{}^{\circ}.$$

**Figure 5:**
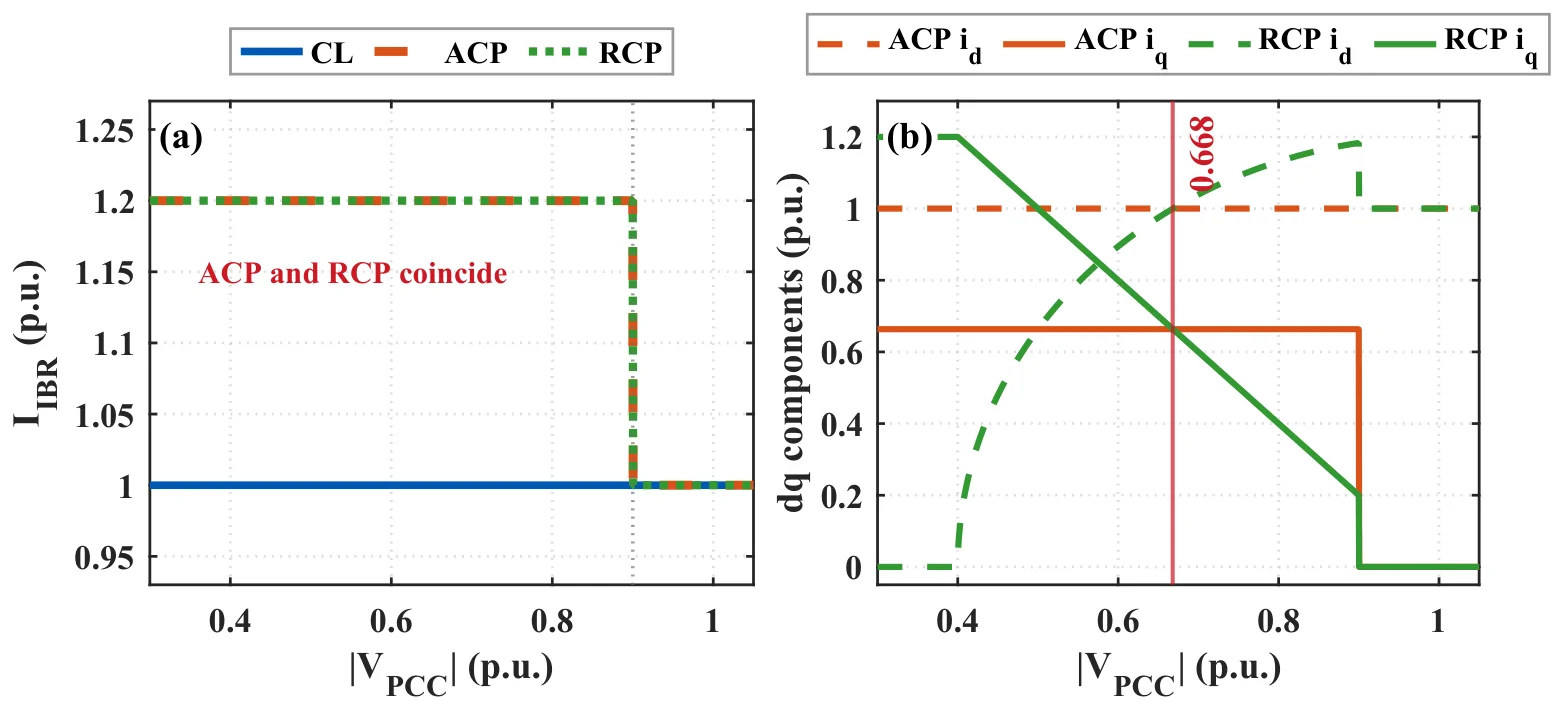
(**a**) Injected current magnitude. (**b**) *d*–*q* components.

**Figure 6:**
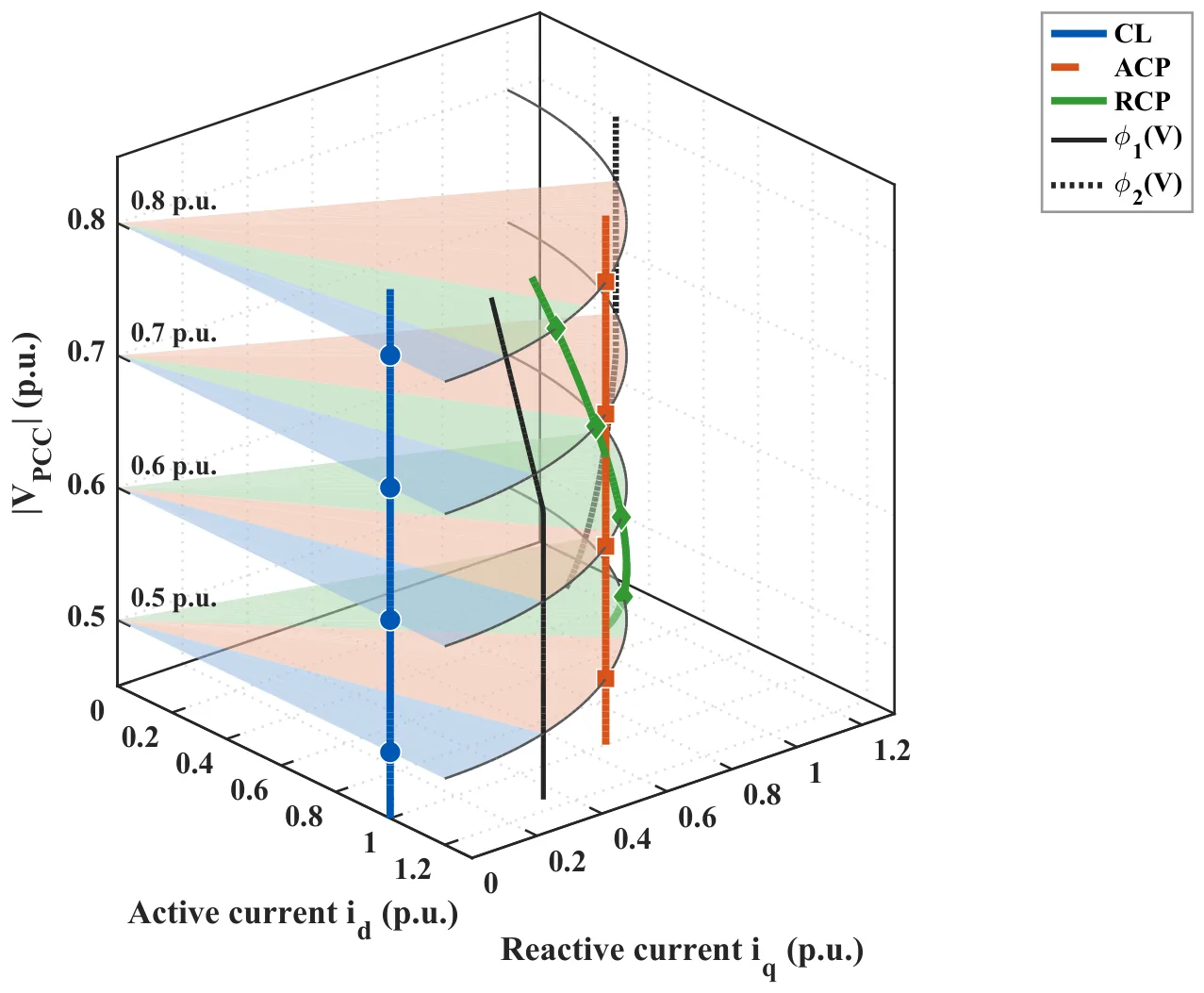
Current fingerprints in the $\left(i_{d},i_{q},|V_{\mathrm{PCC}}|\right)$ space ($k_{q}=2$); shaded sectors are the decision regions.

Under RCP, reactive current follows the voltage support droop of IEEE 2800-2022 and VDE-AR-N 4110 [13,14],

(4) $$i_{q}^{\mathrm{RCP}}=\left\{\begin{array}{cc} 0, & |V_{\mathrm{PCC}}|\;>1\;-\;V_{db},\\ \min\left(k_{q}\left(1\;-\;\right|V_{\mathrm{PCC}}\left|\right)I_{r},\;I_{\max}\right), & |V_{\mathrm{PCC}}|\;\leq 1\;-\;V_{db}, \end{array}\right.\;i_{d}^{\mathrm{RCP}}=\sqrt{I_{\max}^{2}-{\left(i_{q}^{\mathrm{RCP}}\right)}^{2}},$$

so $\varphi^{\mathrm{RCP}}$ rises monotonically as the sag deepens. Figure 5 shows why the angle, not the magnitude, is the discriminating quantity, and the corresponding fingerprint regions in the $\left(i_{d},i_{q}\right)$ plane are illustrated in Figure 6.

The two thresholds that partition the φ axis are the midpoints between adjacent fingerprints of (2)–(4),

(5) $$\varphi_{1}=\frac{1}{2}\left[\varphi^{\mathrm{CL}}+\min\left(\varphi^{\mathrm{ACP}},\varphi^{\mathrm{RCP}}\right)\right],\;\varphi_{2}=\frac{1}{2}\left[\max\left(\varphi^{\mathrm{ACP}},\varphi^{\mathrm{RCP}}\right)+90^{\circ }\right],$$

both evaluated at the measured $|V_{\mathrm{PCC}}|$. With a hysteresis band $\delta_{h}$ that suppresses chatter near a boundary, the decision is

(6) $$\hat{S}=\left\{\begin{array}{cc} {\hat{S}}_{\mathrm{prev}}, & |\varphi \;-\;\varphi_{1}|\;<\delta_{h}\;\mathrm{or}\;\left|\varphi -\varphi_{2}\right|<\delta_{h},\\ \mathrm{CL}, & \varphi <\varphi_{1}-\delta_{h},\\ S_{2}, & \varphi_{1}+\delta_{h}\leq \varphi <\varphi_{2}-\delta_{h},\\ S_{3}, & \varphi \geq \varphi_{2}+\delta_{h}, \end{array}\right.$$

where $\hat{S}$ is the declared mode, ${\hat{S}}_{\mathrm{prev}}$ the decision of the previous cycle, and $S_{2}$ and $S_{3}$ are the two remaining modes ordered by their fingerprint at the measured voltage, so that $S_{2}$ is the one with the smaller angle. The rule is equivalent to assigning the measured angle to the nearest analytical fingerprint at the prevailing voltage. The threshold values are listed in Table 4. Near $|V_{\mathrm{PCC}}|\approx 0.67$ p.u. the ACP and RCP fingerprints coincide, so these two modes cannot be separated by angle in a narrow voltage band.

### 4.3. Ambiguity Band

**Figure 7:**
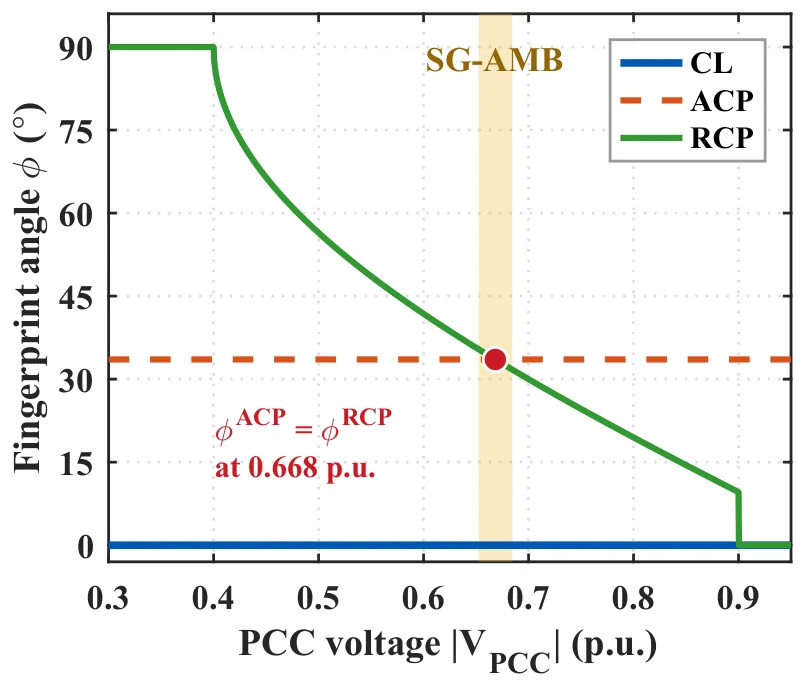
Strategy fingerprints and the ambiguity band (shaded, |Δφ|≤2.33σ).

The coincidence of the ACP and RCP fingerprints is a property of the grid code droop law, not of the measurement, and cannot be removed by better sensing. It is therefore treated as a defined operating condition. The two modes are declared statistically inseparable whenever

(7) $$\left|\varphi^{\mathrm{ACP}}-\varphi^{\mathrm{RCP}}\left(\right|V_{\mathrm{PCC}}\left|\right)\right|\leq 2.33\;\sigma ,$$

the same 1% confidence criterion used to size $\delta_{h}$. For class-0.5 transformers this gives 1.86°, i.e., $|V_{\mathrm{PCC}}|\;\in \left[0.652,\;0.685\right]$ p.u. (Figure 7). The band depends only on the measured voltage, so the relay evaluates it with no extra sensing. Inside it the classifier returns a fourth output, AMB (ambiguous), meaning that the measurement cannot separate ACP from RCP, and activates a group SG-AMB obtained by solving the LP of Section 5 over the union of the ACP and RCP constraint sets. SG-AMB is feasible in both modes; its total operating time exceeds the ACP and RCP optima by 2.4% and 5.2%, respectively, and this cost applies only inside the band. CL remains far from the crossover and is always separable.

### 4.4. Pre-Fault Loading and Mixed Strategies

**Figure 8:**
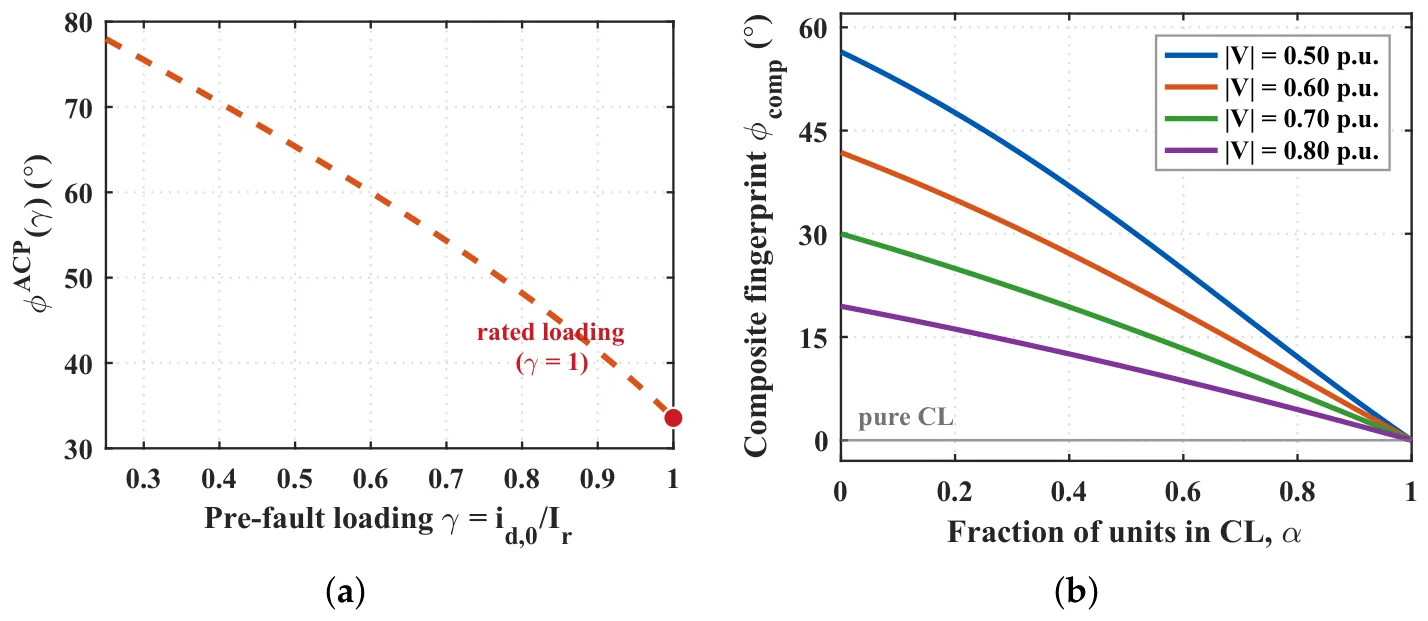
(**a**) ACP fingerprint versus pre-fault loading γ. (**b**) Composite fingerprint of a CL/RCP mix.

The fingerprints above assume rated active current before the fault. With the pre-fault current written as $i_{d,0}=\gamma I_{r}$, γ∈(0,1], the ACP fingerprint becomes

(8) $$\varphi^{\mathrm{ACP}}\left(\gamma \right)=\arctan\frac{\sqrt{I_{\max}^{2}-\gamma^{2}I_{r}^{2}}}{\gamma I_{r}},$$

which moves from 33.6° at γ=1 to 48.2° at γ=0.8 and 60.0° at γ=0.6 (Figure 8a), an order of magnitude more than the hysteresis band. The value of γ is available as the pre-fault current magnitude held in the relay’s one-cycle phasor buffer. The threshold table is therefore indexed by $\left(|V_{\mathrm{PCC}}|,\gamma \right)$ instead of $|V_{\mathrm{PCC}}|$ alone, which accommodates the full irradiance range without telemetry or communication.

If a fraction α of the units on a collector bus operates in mode A and the remainder in mode B, the relay measures the vector sum of the two injections, and the composite angle is

(9) $$\varphi_{\mathrm{comp}}\left(\alpha \right)=\arctan\frac{\alpha \;i_{q}^{A}+\left(1-\alpha \right)\;i_{q}^{B}}{\alpha \;i_{d}^{A}+\left(1-\alpha \right)\;i_{d}^{B}}.$$

Numerator and denominator are convex combinations, so the composite angle lies strictly between the two pure fingerprints for every α∈(0,1) (Figure 8b). The classifier then returns the dominant mode, the worst case is confined to α≈0.5 where either neighboring group is approximately appropriate, and the coordination penalty is bounded by the corresponding off-diagonal entry of the miscoordination matrix evaluated in Section 6.3. Verifying the CTI margins for mixed operation still requires dedicated fault studies (Section 8.5).

### 4.5. Cessation, Asymmetrical Faults and the Sensing Vector

When the PCC voltage falls below $V_{MC}$ and the inverter current falls below $\varepsilon_{I}I_{r}$, where $\varepsilon_{I}=0.10$ is the current-detection threshold, the relay infers MC within one 4 ms cycle:

(10) $$\hat{S}=\mathrm{MC}\;\mathrm{if}\;\left|V_{\mathrm{PCC}}\right|<V_{MC}\;\mathrm{and}\;I_{\mathrm{IBR}}<\varepsilon_{I}I_{r}.$$

An asymmetrical fault activates SG-AS when $|I_{2}\left|/\right|I_{1}|\;>0.20$, and the detectability constraint $I_{f,\min}/I_{p}\geq 1.5$ is enforced for three-phase, line-to-line (LL) and single-line-to-ground (SLG) faults.

Both thresholds follow from physical limits rather than from the results. A balanced fault produces no negative sequence current, and the residual $|I_{2}\left|/\right|I_{1}|$ under balanced conditions is set by load unbalance and transformer error, which IEC 61000-2-2 [52] limits to 2% for the supply voltage and which stays below about 5% in practice. An SLG fault on a solidly earthed feeder gives $I_{2}≃I_{1}$ and an LL fault gives $|I_{2}\left|/\right|I_{1}|\;\to 1$; with fault resistance up to 40Ω the ratio on the studied network does not fall below 0.45. The value 0.20 is thus about four times above the balanced ceiling and more than twice below the asymmetrical floor. The factor 1.5 is the pickup security margin of IEEE C37.112 practice [53].

The decision therefore rests on one sensing vector of quantities the relay already computes,

(11) $$z={\left[\;\varphi ,\;I_{\mathrm{IBR}},\;|I_{2}\left|/\right|I_{1}|\;\right]}^{⊤}.$$

The angle resolves CL, ACP, RCP and, where it cannot, the AMB declaration; the magnitude resolves MC, where the angle is undefined because there is no current; and the sequence ratio resolves asymmetry, which the positive sequence angle cannot express. The angle fingerprint governs the CL/ACP/RCP/AMB decision; MC and AS use standard measurements within the same decision structure. Table 5 lists the resulting groups.

## 5. Coordination Problem

The relays use the IEC 60255-151 standard-inverse characteristic [48],

(12) $$t_{\mathrm{op}}=\mathrm{TMS}\cdot \frac{0.14}{{\left(I_{f}/I_{p}\right)}^{0.02}-1},\;\mathrm{TMS}\in \left[0.05,\;1.10\right],$$

where $t_{\mathrm{op}}$ is the operating time in seconds, TMS the time multiplier setting, $I_{f}$ the fault current seen by the relay and $I_{p}$ its pickup current (Table 3). Selectivity requires, for every primary–backup pair *k*,

(13) $${\mathrm{CTI}}_{k}=t_{b,k}-t_{p,k}\geq {\mathrm{CTI}}_{\min}=0.30\;s,\;k=1,\ldots ,K,$$

where $t_{p,k}$ and $t_{b,k}$ are the primary and backup operating times and K=10 for OM1 and OM5 and K=12 for OM2–OM4, in which the four DOCRs at the PV interconnections are in service. The margin covers a breaker operating time of about 80 milliseconds (ms) and a relay reset allowance of about 200 ms.

For each strategy *S*, the TMS vector $x^{\left(S\right)}={\left[{\mathrm{TMS}}_{1},\ldots ,{\mathrm{TMS}}_{N}\right]}^{⊤}$, N=16, minimizes the total operating time over all primary–backup pairs,

(14) $$\min_{x^{\left(S\right)}}F^{\left(S\right)}=\sum_{k=1}^{K}\left(t_{p,k}^{\left(S\right)}+t_{b,k}^{\left(S\right)}\right),$$

subject to (13), $0.05\leq {\mathrm{TMS}}_{i}\leq 1.10$ and $0.05\leq t_{\mathrm{op},i}\leq 3.00$ s.

The pickup currents are fixed hardware settings and only the TMS vector is optimized, so for a given fault current set the operating time of (12) is

(15) $$t_{\mathrm{op},i}={\mathrm{TMS}}_{i}\;\kappa_{i},\;\kappa_{i}=\frac{0.14}{{\left(I_{f,i}/I_{p,i}\right)}^{0.02}-1},$$

with $\kappa_{i}$ a constant. The objective (14) is therefore linear in $x^{\left(S\right)}$, and each coordination constraint is the linear inequality

(16) $$\kappa_{p,k}\;{\mathrm{TMS}}_{p,k}-\kappa_{b,k}\;{\mathrm{TMS}}_{b,k}\leq -{\mathrm{CTI}}_{\min},\;k=1,\ldots ,K.$$

The remaining bounds are box constraints on the same variables. The coordination sub-problem is thus an LP, whose global optimum is obtained deterministically. All setting groups in this paper are computed in this way and are therefore independent of solver tuning and exactly reproducible. SG-AMB is the solution of the same LP over the union of the ACP and RCP constraint sets. Population-based solvers, which need an unconstrained fitness, are retained only as a benchmark (Section 6.2); for them the constraints are enforced by the quadratic penalty $\lambda \sum_{k}\max{\left(0,{\mathrm{CTI}}_{\min}-{\mathrm{CTI}}_{k}\right)}^{2}$ with $\lambda =10^{8}$. The weight is set three orders of magnitude above the largest attainable objective value, so that any constraint violation dominates the fitness and no infeasible point can be ranked above a feasible one; values from $10^{6}$ to $10^{10}$ gave the same ranking.

To quantify the degradation when the relay holds the group optimized for $S_{0}$ while the inverter is in $S^{\prime }$, where $x^{\left(S_{0}\right)}$ denotes the TMS vector of that group, the strategy switching miscoordination index is

(17) $$M\left(S_{0}\to S^{\prime }\right)=\frac{1}{K}\sum_{k=1}^{K}\max\left(0,{\mathrm{CTI}}_{\min}-{\mathrm{CTI}}_{k}^{\left(S^{\prime }\right)}\left(x^{\left(S_{0}\right)}\right)\right).$$

**Figure 9:**
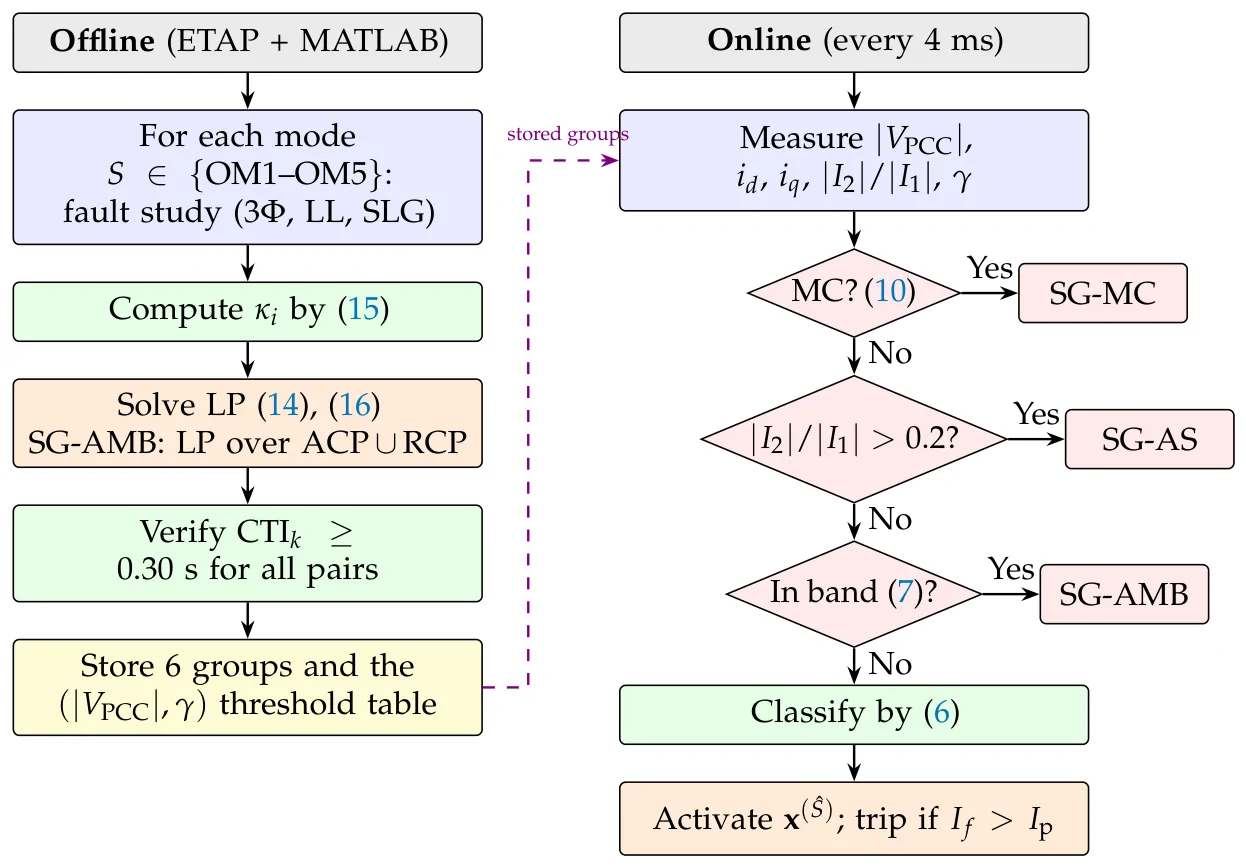
Offline and online stages of the proposed scheme.

Here ${\mathrm{CTI}}_{k}^{\left(S^{\prime }\right)}\left(x^{\left(S_{0}\right)}\right)$ is the margin of pair *k* when the relay holds the group of $S_{0}$ while the inverter operates in $S^{\prime }$. The complete offline/online workflow is summarized in Figure 9.

The six stored groups are those of Table 5: one for each inverter mode that the relay can distinguish (CL, ACP and RCP), one for the ambiguity band, one for cessation and one for asymmetrical faults. No further group is required, because every condition the sensing vector can report maps onto one of these six. In the online branch the relay loads the TMS vector $x^{\left(\hat{S}\right)}$ of the selected group and trips when the measured current exceeds the pickup current, so the classification changes the operating time of the element and never the pickup decision itself.

## 6. Results: CIGRE MV Benchmark

Using an Intel Core i7-12700 (Intel Corporation, Santa Clara, CA, USA) workstation with 32 GB of RAM, the fault studies were executed in ETAP, while the corresponding setting groups were optimized in MATLAB R2024a (MathWorks, Natick, MA, USA) with the linprog function.

### 6.1. Optimal Setting Groups

The LP-optimal TMS vectors are provided in Table 6, whereas Table 7 summarizes the fault current levels and the associated coordination margins for each relay pair under all operating modes. Every pair satisfies the 0.30 s margin in every mode, and the minimum CTI of each LP solution is exactly 0.300 s, as expected of a vertex optimum. Only F9 and F10 retain larger margins, set by the impedance geometry of those pairs. Relays near the PV connections, OCR_4_, OCR_8_ and OCR_12_, show the largest TMS changes across modes.

**Figure 10:**
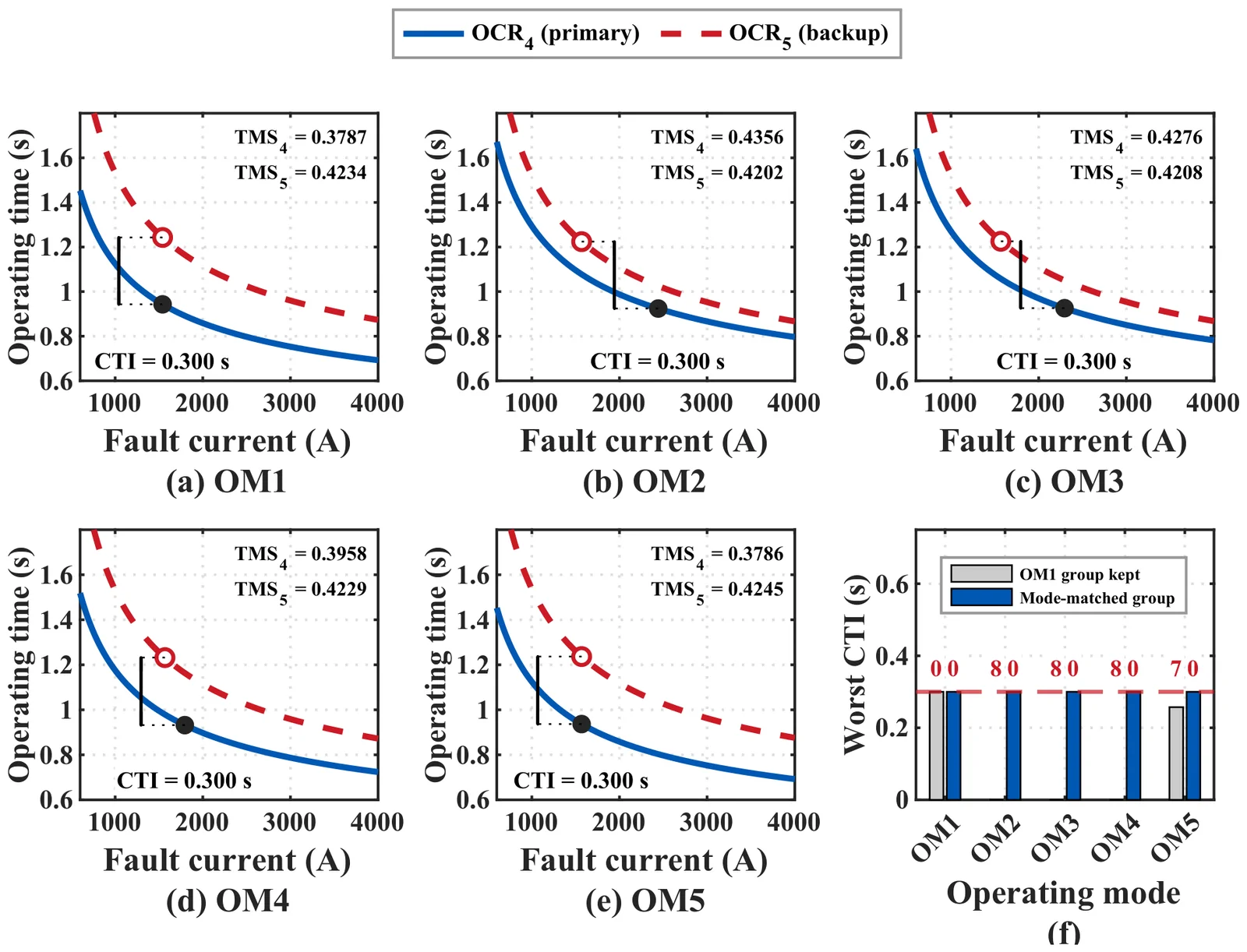
OCR_4_/OCR_5_ coordination at F4. (**a**–**e**) OM1–OM5. (**f**) Worst CTI with the OM1 group retained.

Figure 10 traces the OCR_4_/OCR_5_ pair at F4. The primary current rises from 1541 A in OM1 to 2442 A under CL, while the backup current stays near 1568 A; the matched groups hold the margin at exactly 0.30 s in every mode. If the OM1 group is retained (Figure 10f), eight pairs lose their margin in each inverter-active mode and seven in OM5. Table 8 summarizes the five groups, in which $F^{⋆}$ denotes the optimal value of the total operating time of (14).

**Figure 11:**
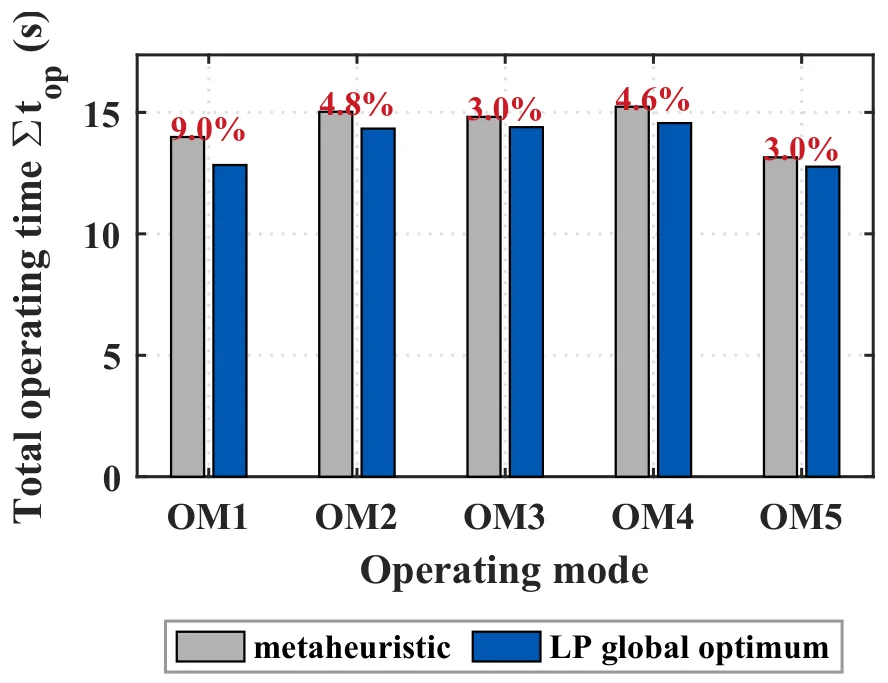
Original metaheuristic solutions versus the LP optimum.

The last column compares the exact optimum with the metaheuristic vectors of the original submission (Figure 11). The gaps of 3–9% show that the earlier search had not converged to the optimum. Re-evaluated with the pickup settings listed in Table 3, the earlier OM1 vector gives a CTI of merely 0.052 s at F4, violating the minimum threshold. That vector was therefore infeasible and has been superseded by the LP-generated setting groups.

### 6.2. Solver Benchmark

The rime optimization algorithm (RIME) [54], GA [55], PSO [56], WOA [57] and GWO [21] were run 30 times each on all five modes, with a common population, evaluation budget and feasible initial point, and compared with the exact LP optimum of Section 6.1 (Table 9). Every solver ends within 0.07% of the LP optimum in every mode, and none of the 750 runs reproduces it exactly; the closest single run, obtained with PSO, stops $1.1\times 10^{-6}$% above it. The comparison is therefore not between competing solvers but between each solver and the exact solution used in this paper, which no solver improves on.

The contribution of the search itself is small. The constructive feasible seed used to start every solver, obtained by iterative relaxation of the CTI constraints, already lies within 0.064% of the optimum, so the iterations that follow recover only the remaining 0.06%. Started without that seed, the best solver stops 18% above the optimum and the others do not reach a feasible point within the same evaluation budget. What distinguishes the solvers on this problem is thus the initialization rather than the search strategy, which is the reason the setting groups of this paper are taken from the LP solution and the metaheuristics are reported only as a reference. A Friedman test over the 150 pooled runs per solver rejects equal performance ($p<10^{-100}$), and Wilcoxon signed-rank post hoc tests confirm that the ordering is systematic. The differences, however, are at most 0.06% of the total operating time, about 9 ms over sixteen relays, and have no practical significance.

### 6.3. Miscoordination Under Strategy Switching

Table 10 gives $M(S_{0}\to S^{\prime })$ of (17) for a single-setting scheme that keeps the group of $S_{0}$. Off-diagonal entries reach $6.39\times 10^{-2}$ s, with the MC row worst because the fault current distribution without inverter infeed differs most. With ideal classification the proposed scheme exercises only the diagonal, so M=0; the effect of realistic classification errors is quantified in Section 6.5. Table 11 compares the scheme with the single-setting reference and with EVPM [33].

### 6.4. Line-to-Line and Single-Line-to-Ground Faults

The groups of Table 6 were derived from three-phase fault currents. To check that they remain valid for asymmetrical faults, LL and SLG faults were applied at every fault location and the matched three-phase group was kept unchanged. A three-phase, LL, SLG and (LLG) short-circuit study to IEC 60909 [47] (minimum currents, c=1.0) was run in ETAP on the network without inverter infeed. It reproduces the three-phase currents of Table 7 within 3% along the feeders and gives, at every location, $I_{\mathrm{LL}}/I_{3\Phi }=0.866$ and $I_{\mathrm{SLG}}/I_{3\Phi }$ between 0.77 and 0.78 on the feeder buses, 0.82 at B2, 0.70 at B13 and 1.02 at the solidly earthed secondaries B1 and B12. These location-specific ratios were applied to the three-phase currents of each pair. For the utility-only modes OM1 and OM5 this is the complete answer. In the inverter-active modes the utility part is scaled in the same way. The inverter part was taken from the EMT model of Section 6.9: for PCC voltages below 0.9 p.u. the positive sequence inverter current in LL and SLG faults is within 2% of its three-phase value, because the current limit is active and no zero-sequence current flows behind the Dyn step-up transformers. The inverter part is therefore kept at its three-phase value for both fault types. Table 12 gives the result. Without inverter infeed the smallest current multiple is 8.0 (SLG) and the minimum CTI is 0.314 s for LL and 0.323 s for SLG faults, with no violation (Table 12). With the inverters active the smallest multiple is 6.0 (OM4, directional pairs), well above the detectability limit of 1.5, and no violation occurs at any location. The directional pairs remain at 0.300 s. Because the standard-inverse curve is steeper at lower multiples, the reduced currents lengthen backup times more than primary times, so asymmetrical faults do not erode the margin. A union LP over the three-phase, LL and SLG constraint sets returns the same vectors as the three-phase LP; no separate asymmetrical group is needed for phase elements, and SG-AS coincides with the MC group. SLG faults on resistance-earthed feeders, where earth-fault elements govern, are outside the present scope.

### 6.5. Classification Under Measurement Noise

The classifier was evaluated by Monte Carlo simulation of its analytical model: for each mode the fingerprint $\varphi^{S}\left(\right|V_{\mathrm{PCC}}\left|\right)$ was perturbed by zero-mean Gaussian angular noise with σ=0.8° over $|V_{\mathrm{PCC}}|\;\in \left[0.3,0.9\right]$ p.u. ($3\times 10^{5}$ trials per mode).

**Figure 12:**
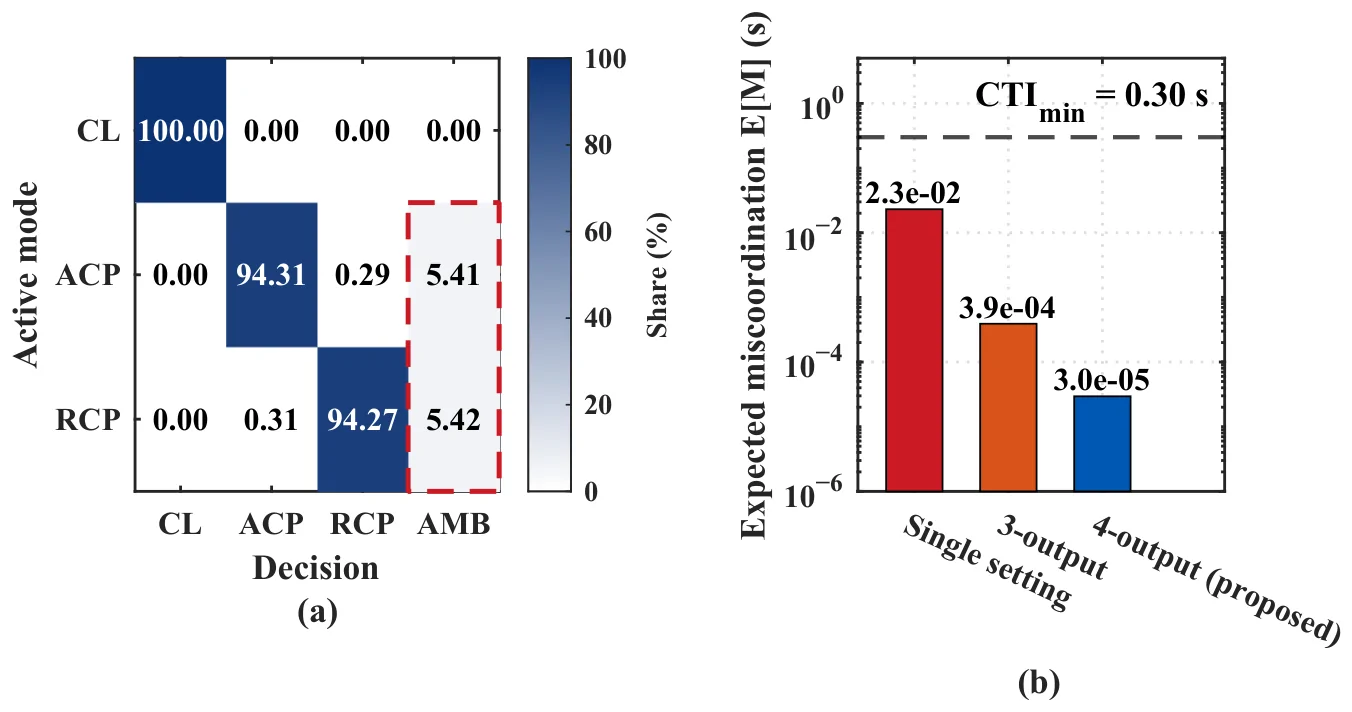
(**a**) Decision distribution; the red dashed box marks the ACP and RCP cases assigned to the ambiguous output (AMB), for which group SG-AMB is placed in service. (**b**) Expected residual miscoordination.

The classifier has four outputs: CL, ACP, RCP and AMB. AMB is a valid decision because it activates a group feasible for both candidate modes, so the figure of merit is whether the group placed in service preserves the margin for the mode actually active. CL is recovered without error at every voltage (Table 13, Figure 12a). For ACP and RCP about 5.4% of cases fall inside the band and are served by SG-AMB; genuine misclassification outside the band is 0.29% (ACP read as RCP) and 0.31% (RCP read as ACP). The activated group is coordination-valid in 99.80% of all cases.

When the active mode is $S^{\prime }$ but the group of $S_{0}\neq S^{\prime }$ is activated, the resulting miscoordination is the entry $M(S_{0}\to S^{\prime })$ of Table 10, so the expected residual is

(18) $$E\left[M|S^{\prime }\right]=\sum_{S_{0}\neq S^{\prime }}\mathrm{Pr}\left(\hat{S}=S_{0}|S^{\prime }\right)\;M\left(S_{0}\to S^{\prime }\right).$$

Decisions inside the band contribute nothing, because SG-AMB is feasible for both modes. Evaluating (18) with Table 13 gives $E\left[M\right]\leq 3.0\times 10^{-5}$ s for the worst mode, 0.01% of the 0.30 s margin and almost three orders of magnitude below the $2.31\times 10^{-2}$ s of a single-setting scheme (Figure 12b). A three-output classifier, which forces the ambiguous cases into ACP or RCP, gives $3.9\times 10^{-4}$ s, so the ambiguity band gives a thirteen-fold reduction. The remaining errors are ACP↔RCP confusions whose penalty is itself small (0.95 and $1.01\times 10^{-2}$ s), because nearly identical fingerprints imply nearly identical settings, and the cases where the fingerprints are closest are exactly those removed by SG-AMB. MC and AS are detected by magnitude and sequence ratio, not by angle, and are not confused with the angle-based modes under normal measurement noise.

**Figure 13:**
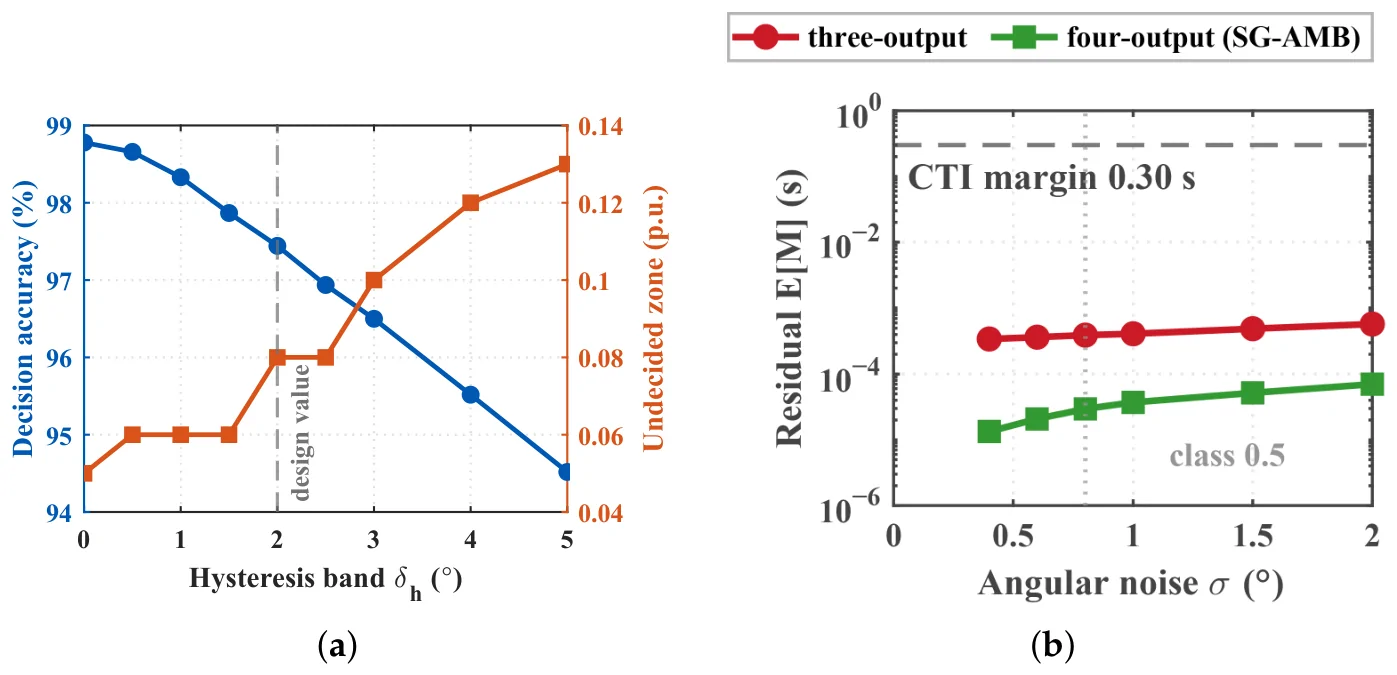
(**a**) Effect of the hysteresis band. (**b**) Residual miscoordination versus noise level.

The hysteresis band trades static discrimination against immunity to chatter. Without it the decision accuracy is 98.7%; it falls to 98.3%, 97.5% and 96.5% for $\delta_{h}=1^{\circ }$, $2^{\circ }$ and $3^{\circ }$, because a wider band enlarges the region without a confident decision (Figure 13a). Once that region is served by SG-AMB the penalty no longer reaches coordination, so $\delta_{h}$ can be chosen for transient immunity alone. The band width scales with σ by construction (7), so a noisier measurement widens the band rather than producing wrong settings. Table 14 and Figure 13b show that the residual does not exceed $7\times 10^{-5}$ s up to σ=2.0°, 2.5 times the class-0.5 limit.

*Detection latency.* From the settling model of Section 2, the current reaches its steady state within about 0.6 ms, after which the classifier decides once per 4 ms cycle. The resulting latency has a mean of 2.6 ms and a 95th percentile of 4.4 ms, two orders of magnitude below the relay operating times.
*Parameter sensitivity.* The RCP fingerprint depends on the reactive gain $k_{q}$, with $\partial \varphi^{\mathrm{RCP}}/\partial k_{q}\approx 25^{\circ }$ per p.u./p.u. at $|V_{\mathrm{PCC}}|\;=0.60$ p.u. For ±20% deviations in $k_{q}$, $\varphi_{1}$ stays within 16.1–16.8° and $\varphi_{2}$ shifts by at most 5.7° (Table 15); a Monte Carlo run with uniform ±20% gain error gives 3.1% misclassification, reduced below 1% by measuring $k_{q}$ at commissioning.

### 6.6. PLL Bandwidth and Grid Strength

The fingerprint assumes that the PLL has re-locked to the post-fault voltage when the decision is taken. A fault produces a phase jump at the PCC that grows as the grid weakens, i.e., as the short-circuit ratio (SCR) falls, and the PLL removes it at a rate set by its bandwidth. A first estimate follows from a second-order PLL of damping ratio ζ=0.707 and natural frequency $\omega_{n}=2\pi f_{\mathrm{PLL}}/2.06$, where $f_{\mathrm{PLL}}$ is the loop bandwidth, with the initial jump $\Delta \theta_{0}=\arctan\left[0.36/\left(0.5\;\mathrm{SCR}\right)\right]$. The settling time was then obtained from the EMT model of Section 6.9, in which an SRF-PLL of the same bandwidth drives the inverter and the angle between the PLL output and the measured terminal voltage is recorded for a three-phase fault (RCP, 5 Ω). Table 16 gives the time after which this error remains below $\delta_{h}=2^{\circ }$. The EMT values are two to four times the analytical estimate, because the voltage sag, the transition to the current limit and the droop response take place in the same interval. A 50 Hz PLL settles in 34–54 ms for SCR ≥2, a 100 Hz PLL settles in about 20 ms for SCR ≥3, is not stable at SCR =2 and needs 185 ms at SCR =1.5, and a 20 Hz PLL needs 79–187 ms. The first mode decision is therefore held for the settling time that corresponds to the SCR at the PCC, which is known from the interconnection study, and the pre-fault group (SG-CL) stays in service meanwhile. Because $t_{\mathrm{op}}=\mathrm{TMS}\;\kappa$ and both groups see the same current, the trip-time displacement is $\Delta \cdot |{\mathrm{TMS}}_{\mathrm{right}}/{\mathrm{TMS}}_{\mathrm{wrong}}-1|$, where Δ is the duration for which the wrong group is in service and ${\mathrm{TMS}}_{\mathrm{right}}$ and ${\mathrm{TMS}}_{\mathrm{wrong}}$ are the settings of the correct and of the held group. With the largest TMS ratio between SG-CL and SG-RCP, 9.1% (OCR_4_), the displacement is at most 4.9 ms (1.6% of the margin) for a 50 Hz PLL and SCR ≥2, and 17 ms (5.7%) for the slowest stable case (20 Hz, SCR =1.5). The validity range is thus SCR ≥1.5 for a 20 Hz PLL, SCR ≥2 for 50 Hz and SCR ≥3 for 100 Hz.

### 6.7. Current Transformer Saturation

**Figure 14:**
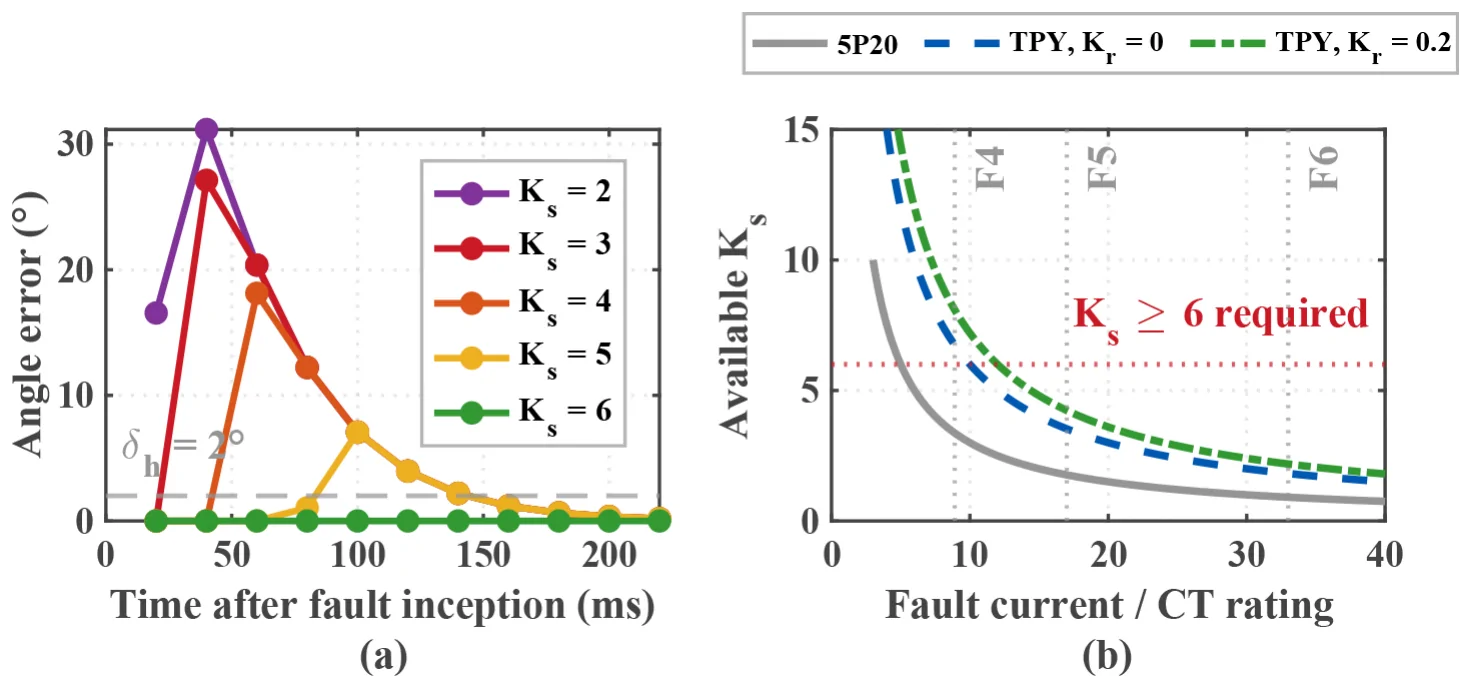
CT saturation. (**a**) Angle-error decay. (**b**) Available flux factor versus fault current multiple.

At the close-in faults of this network the current reaches 33 times the CT primary rating, where the dominant error is core saturation driven by the decaying dc component. A flux-limited core model in the sense of IEEE C37.110 [58] is used, with primary current $i\left(t\right)=I[\sin\left(\omega t+\psi \right)-\sin\left(\psi \right)e^{-t/T}]$, where ψ is the fault inception angle and T=(X/R)/ω the dc time constant set by the system X/R ratio; the core flux integrates the secondary current and clamps at saturation, so the core recovers without external reset. Severity is expressed by the ratio $K_{s}$ of available flux capacity to symmetrical flux demand. Symmetrical saturation reduces the fundamental but does not shift its phase; the angle is shifted by the *asymmetry* of the dc offset, so the worst case is inception at voltage maximum with the highest X/R ratio. For $K_{s}\geq 6$ the angle error stays inside the hysteresis band (Figure 14a). At $K_{s}=5$ it peaks at 7.1° and returns below $2^{\circ }$ within one further cycle; at $K_{s}\leq 3$ it reaches 27–$31^{\circ }$ and takes about 140 ms to decay. The same core model, applied in the EMT model to the feeder current of a close-in three-phase fault with four inception angles, gives no angle error for $K_{s}\geq 6$, at most 3.5° for $K_{s}=5$, and $27^{\circ }$ and $38^{\circ }$ for $K_{s}=3$ and 2, with recovery within 134 ms. Figure 14b maps this requirement onto the network. Class 5P20 protection cores do not meet it at any studied location; transient class TPY cores with a transient dimensioning factor $K_{td}=4$ meet it at F4 and are marginal at F5. The practical consequence is limited because the most severe locations, F6 and F12, are terminal faults with no backup pair, and elsewhere a one-cycle wait before the first decision suffices. The scheme therefore presumes transient-class CTs at relays that carry a coordination pair and a first decision one cycle after inception.

### 6.8. Dynamic Verification

**Figure 15:**
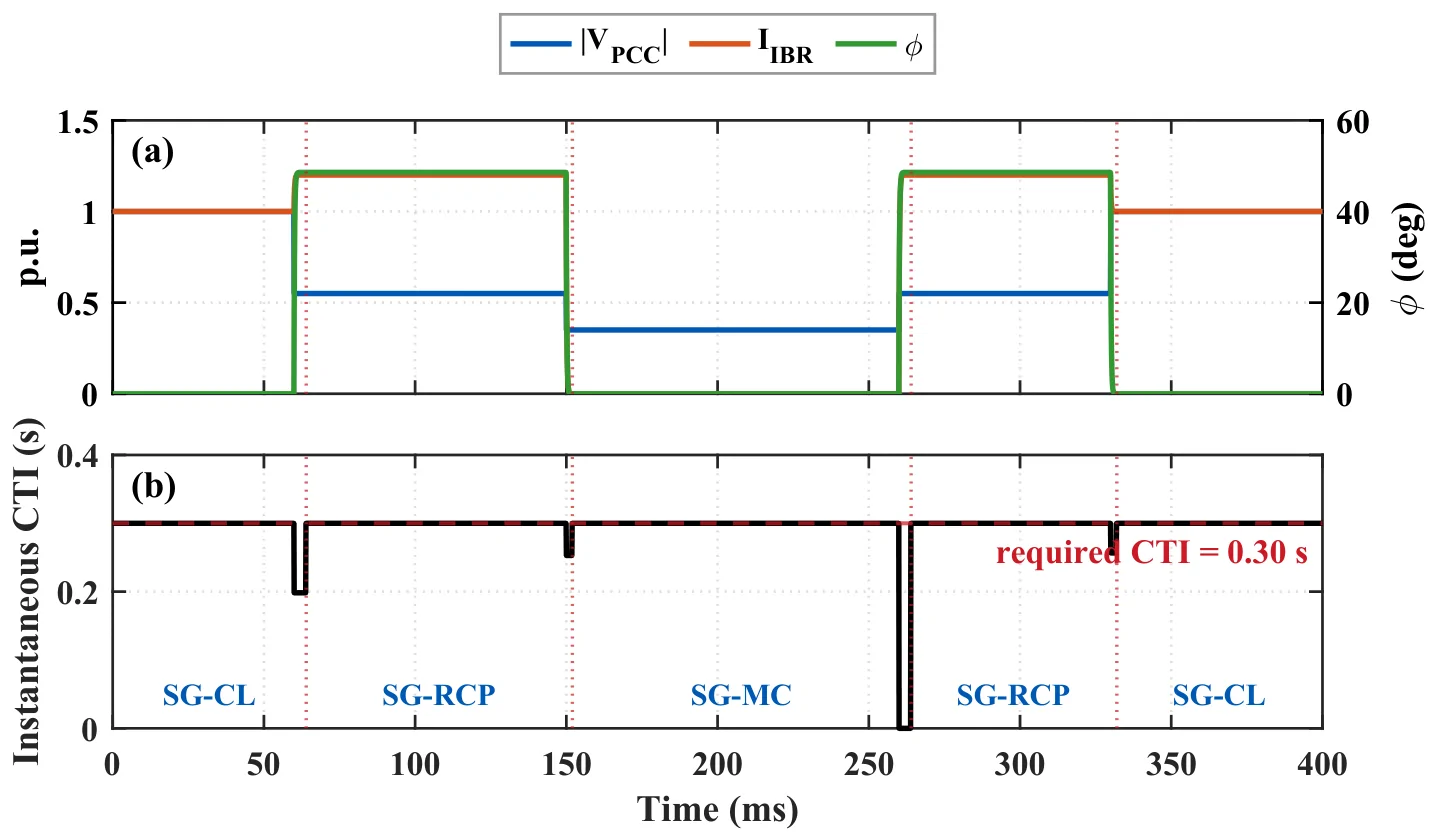
End-to-end response for four mode transitions. (**a**) PCC voltage $|V_{\mathrm{PCC}}|$, inverter current $I_{\mathrm{IBR}}$ and fingerprint angle ϕ. (**b**) Instantaneous CTI margin with the active setting group.

The complete chain of measurement, classification, group activation and resulting margin was run in the time domain through the transitions CL→RCP→MC→RCP→CL, with first-order inner-loop settling, one decision per 4 ms cycle under class-0.5 noise, and the group loaded on the cycle after a decision change (Figure 15). Every transition is detected within one cycle and the switching latency is 4.1 ms. In between, the previous group remains loaded and the instantaneous margin dips for 1.8–3.8 ms per transition. The critical transition is MC→RCP, where the directional pairs briefly lose their margin because the MC group leaves them at minimum TMS. Constraining the MC group to remain feasible for the resumption modes, with the same union formulation as SG-AMB, gives an *infeasible* LP: no single TMS vector satisfies both the cessation and inverter-active cases, so the switching window must be bounded rather than eliminated. Because an inverse-time relay integrates, a mismatch of duration Δ=4.1 ms displaces a trip by at most 13.2 ms over all pairs, 4.4% of the margin, and the window is 1.1% of the fastest backup time (379 ms). Coordination is maintained through the transitions with more than 95% of the margin intact. A closed-loop EMT verification is given in Section 6.9.

### 6.9. Electromagnetic Transient Verification

**Figure 16:**
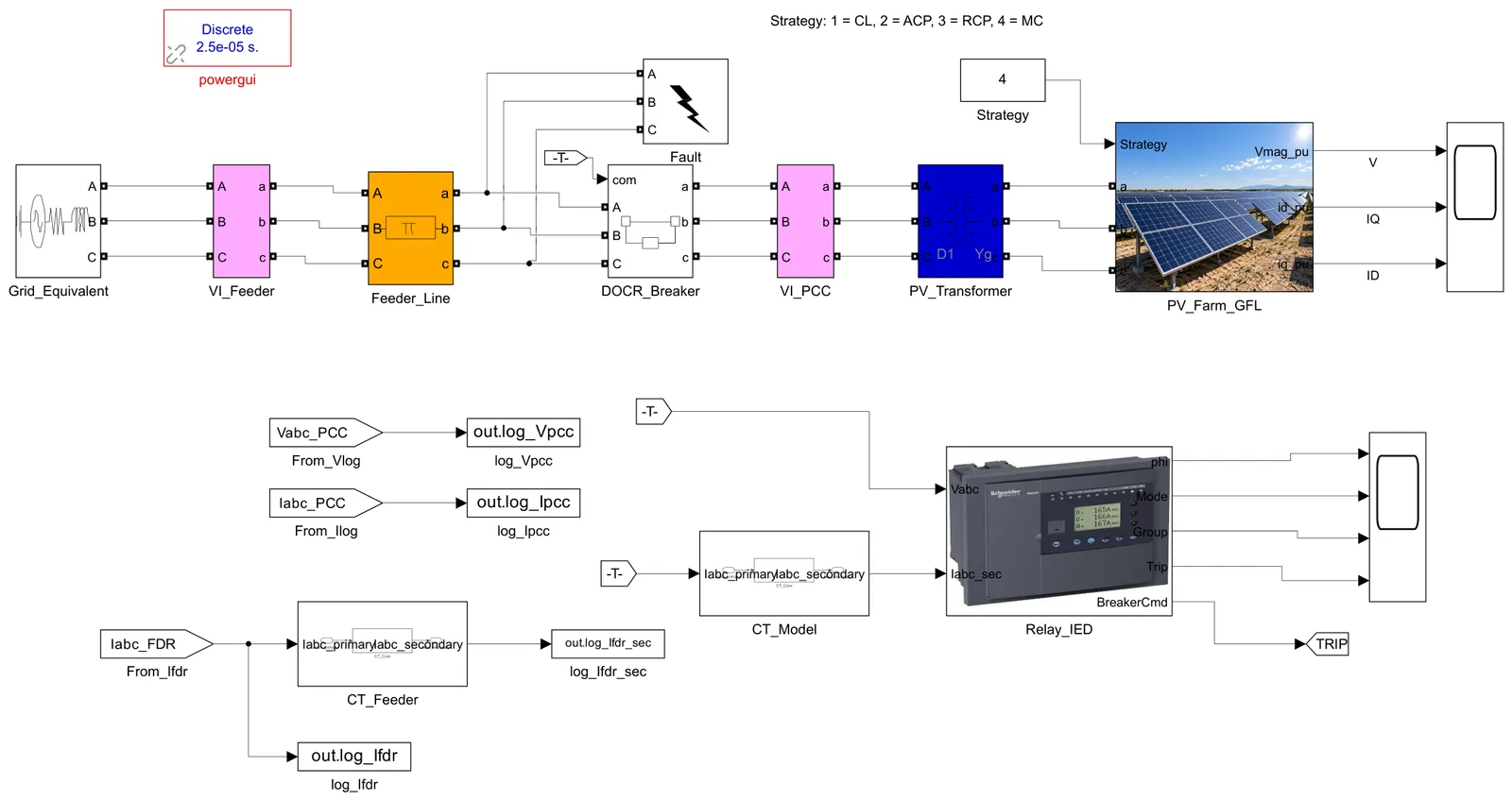
EMT model in MATLAB/Simulink; block colors follow the default Specialized Power Systems library palette.

The sensing layer was verified on an EMT model built in MATLAB/Simulink R2024a (MathWorks, Natick, MA, USA) (Specialized Power Systems, discrete solver, 25 μs step), shown in Figure 16. The model represents the feeder section at the PCC of one PV farm. It contains a Thévenin equivalent of the upstream network with the minimum short-circuit level from ETAP (1541 A, X/R=10), a PI-section feeder with the CIGRE line data, a three-phase fault block, the DOCR breaker and the 20/2 kV Dyn step-up transformer. The PV farm (ten 1.35 MVA units) is an averaged GFL model. It includes an SRF-PLL, a current-reference block that implements CL, ACP, RCP and MC with the parameters of Table 2 and the PCC voltage as droop input, first-order inner current loops, controlled current sources and an RC filter. The relay block contains a flux-limited CT, one-cycle sequence phasor estimation, the classifier of Section 4 executed every 4 ms, and a voltage-supervised IEC 60255 element whose TMS is taken from the active group. A second CT model on the feeder current is used for the saturation study.

**Figure 17:**
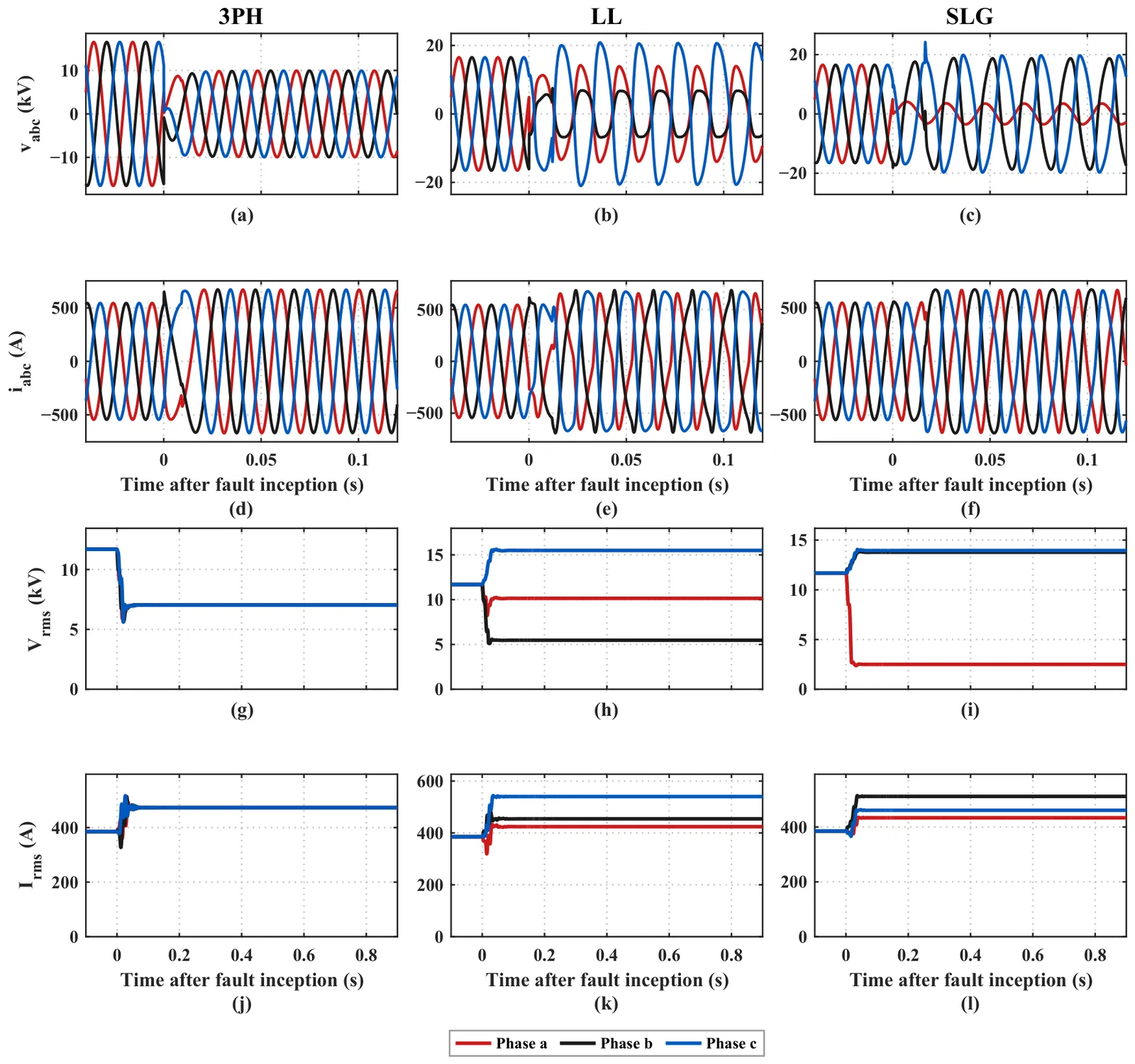
PCC voltages and currents, RCP. (**a**–**f**) Instantaneous values. (**g**–**l**) RMS values.

**Figure 18:**
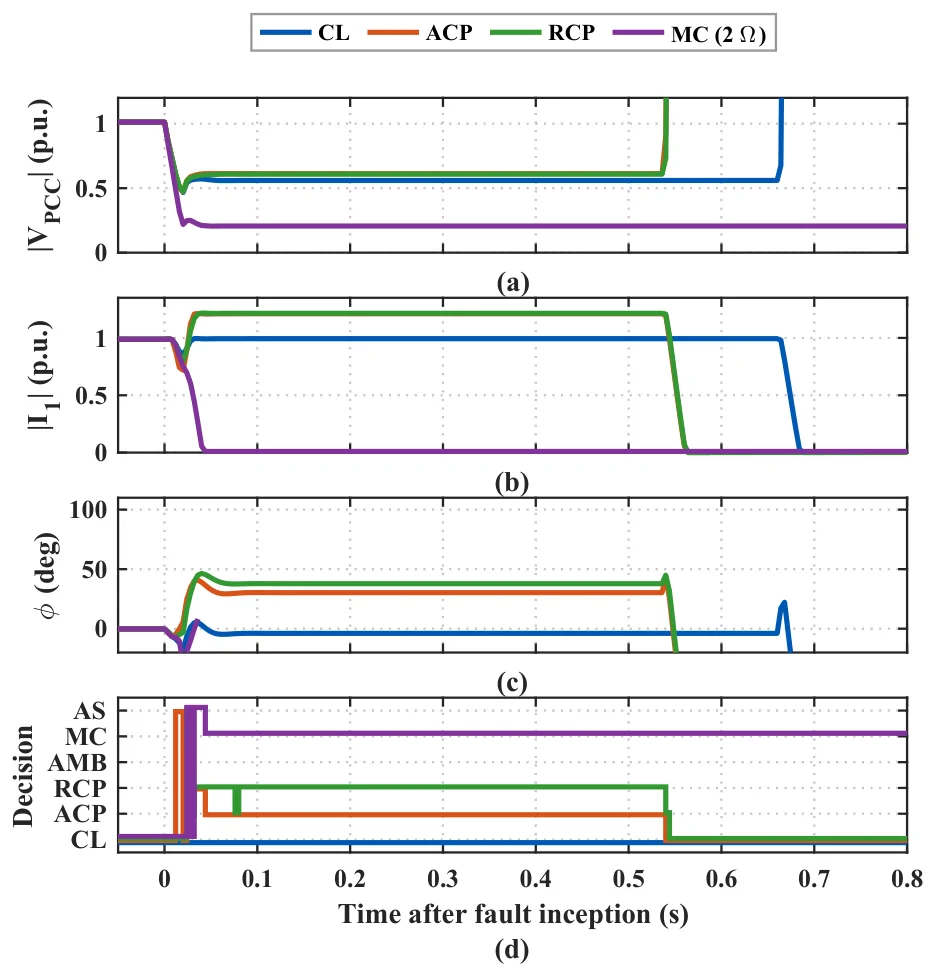
Relay quantities, three-phase fault. (**a**) PCC voltage. (**b**) Current. (**c**) Fingerprint. (**d**) Decision.

Three sets of runs were performed: the four strategies with three-phase, LL and SLG faults and fault resistances $R_{f}$ of 2, 5 and 10 Ω (36 cases, 2 s each), the PLL sweep of Section 6.6, and the CT sweep of Section 6.7. Figure 17 shows the PCC voltages and currents for RCP. In all three fault types the inverter current reaches the 1.2 p.u. limit within one cycle, remains balanced, and falls to zero when the DOCR opens. Figure 18 shows the relay quantities for a three-phase fault. The angle settles at $-4^{\circ }$, $30^{\circ }$ and $38^{\circ }$ for CL, ACP and RCP, the decision is reached within 4–80 ms, and the MC case (2 Ω, $|V_{\mathrm{PCC}}|\;=0.21$ p.u.) is recognized from the loss of current. The trip times, 1.14 s (CL), 0.95 s (ACP) and 0.94 s (RCP) for the 5 Ω fault, agree within 3% with the IEC characteristic of the active group.

**Figure 19:**
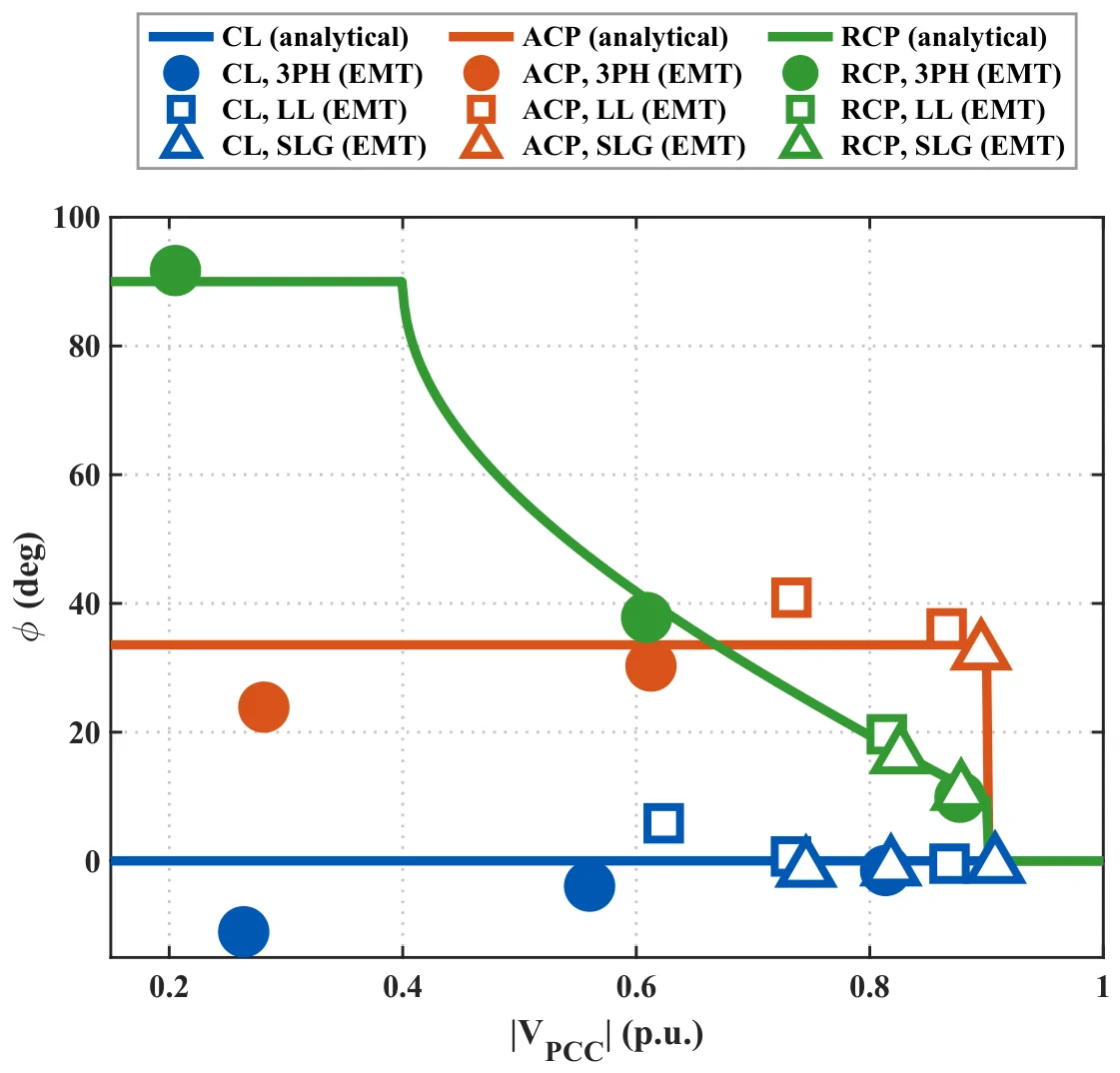
Measured (EMT) and analytical fingerprints. Lines: analytical fingerprints; markers: EMT measurements (colour: operating mode; shape: fault type).

Figure 19 compares the measured fingerprints with the analytical curves of Section 4. For moderate sags, the EMT angle is 1–3^∘^ below the analytical value, mainly because of the filter capacitor and the transformer reactance between the inverter and the relay. For deep sags ($|V_{\mathrm{PCC}}|\;\approx 0.26$ p.u.), the deviation reaches about 10^∘^, which still lies inside the correct decision region. In 32 of the 36 cases, the classifier selected the group that matches the injected current. Three of the other four cases have PCC voltages of 0.89–0.90 p.u., at the edge of the deadband, and one (RCP, LL, 2 Ω) is an ACP/RCP confusion near the crossover, for which the miscoordination penalty is small (Table 10). When the PCC voltage remains above 0.9 p.u., as for the 10 Ω LL and SLG faults, the inverters do not enter fault ride-through and the CL decision is correct. In LL and SLG faults the inverter current contains almost no negative sequence component, so $|I_{2}\left|/\right|I_{1}|$ at the PV-side DOCR stays below 0.17. Asymmetry is therefore detected by the feeder relays, which carry the grid contribution, while the PV-side relay selects its group from the angle.

## 7. Case Study II: Nediba Feeder

**Figure 20:**
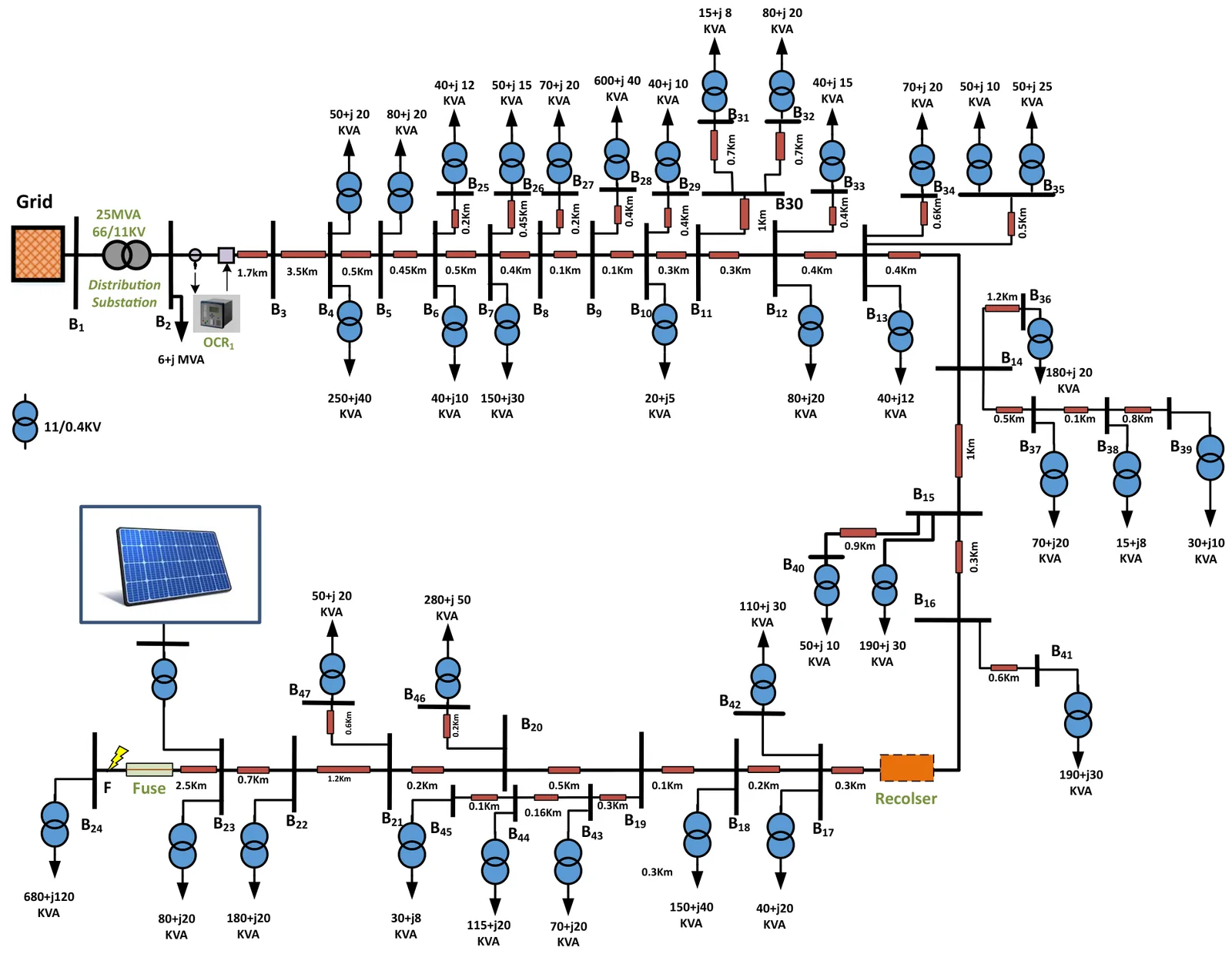
Nediba 11 kV feeder with the 3 MVA PV farm; blue circles denote 11/0.4 kV distribution transformers.

The second case study uses a practical model of the Nediba 11 kV feeder, a rural feeder in Damanhur, Egypt, supplied from the 66/11 kV Khairy substation. It has 47 buses along a 16 km ACSR 70/12 overhead line [37], an OCR at the feeder head, a recloser at 10.65 km and a 100 A fuse at 13.5 km. The original 3 MVA synchronous DG at bus 23 is replaced by a 3 MVA GFL-PV farm scaled from Table 2, and a DOCR is added at the PV connection (Figure 20). The fuse total clearing and minimum melting times follow [37,59]

(19) $$t_{\mathrm{TC}}=ae^{bI}+ce^{dI},\;t_{\mathrm{MM}}=t_{\mathrm{TC}}-0.10\;s,$$

with *I* the fuse current in amperes, a=45.68, b=−0.006984, c=1.681 and d=−0.00145. The recloser and the feeder relay use the IEC very-inverse characteristic with time-dial setting (TDS), $t=\mathrm{TDS}\cdot 13.5/(I/I_{p}-1)$. The recloser phase element has $I_{p}=160$ A with fast and slow TDS of 0.0998 and 0.2619, its earth element has $I_{p}=32$ A and a fast TDS of 3.6442, and the relay has $I_{p}=390$ A and TDS =0.1153 [59]. The required margin Δt between the fuse minimum melting time of (19) and the recloser fast curve, and between the recloser slow curve and the relay, is 0.20 s.

**Figure 21:**
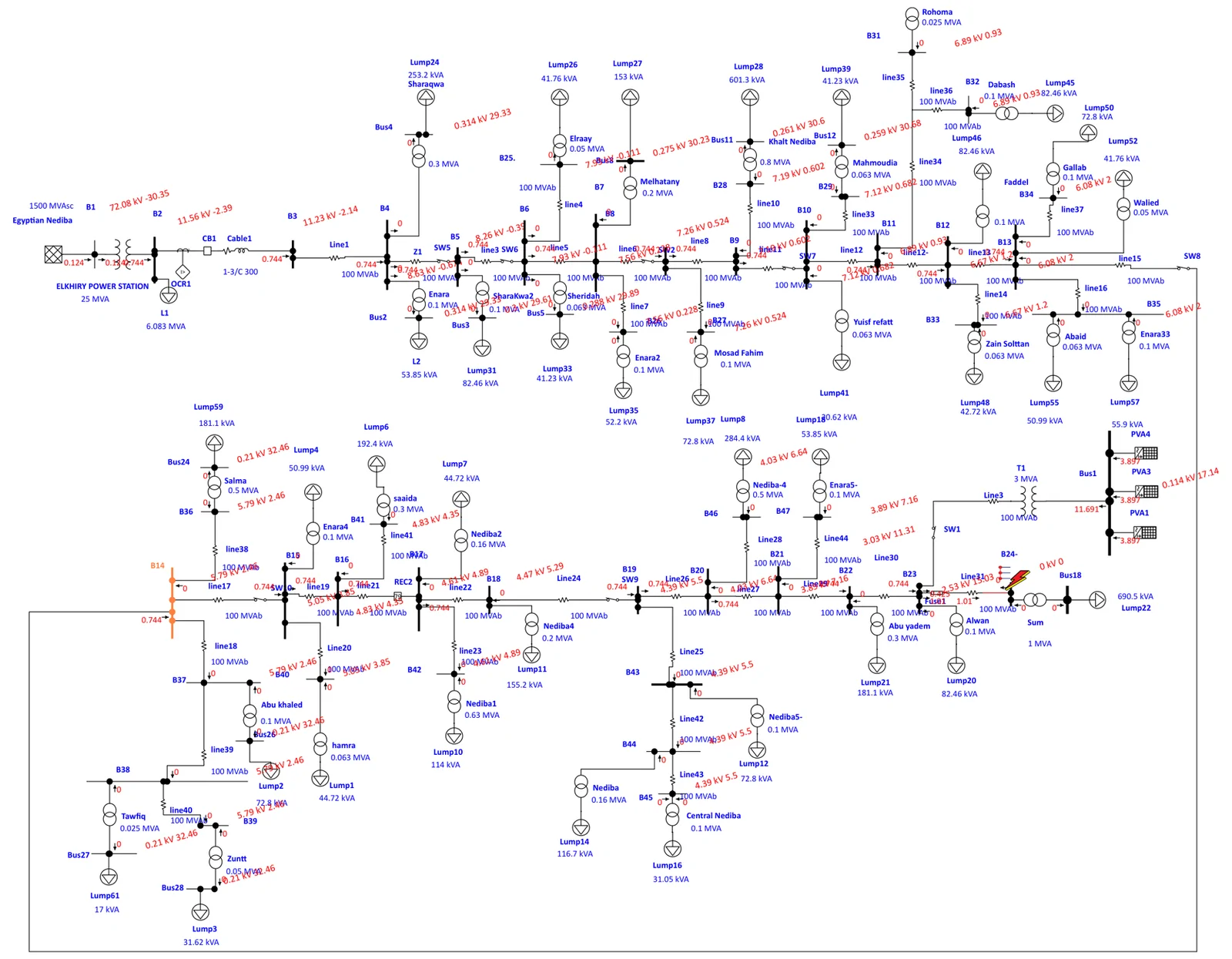
ETAP model of the Nediba feeder with the PV farm at bus 23; colors follow ETAP’s default load-flow display and the orange marker identifies the recloser.

The feeder was modeled in ETAP (Figure 21) with IEC 60909 (c=1.0, static loads). Without PV, the model reproduces the fault currents of [59]: 846 A and 710 A for three-phase faults at F1 and F2 (852–860 A and 700–716 A in the reference), and 395 A and 329 A for SLG faults (366–403 A and 321–359 A). The study covers three fault points: F1 just downstream of the fuse, where the fuse current is largest; F2 at the end of the fused lateral, 2.5 km further; and F3 just downstream of the recloser, which tests the recloser–relay pair. Three-phase, LL and SLG faults with $R_{f}=0$, 5 and 10 Ω are applied in each mode, giving 18 fuse–recloser cases per mode. The PV farm is represented as a positive sequence current source at bus 23, with the mode-dependent current of Section 4 and no zero-sequence path through its delta-connected 11 kV winding, and the network part is taken from the ETAP Thévenin impedances. Table 17 lists the currents for solid faults.

Because the PV farm lies between the recloser and the fuse, it raises the fuse current while the recloser current stays at or below its no-PV value. The increase is largest for three-phase faults at F1 (up to 1034 A under ACP), where the voltage at the inverter terminals falls below 0.1 p.u.; the RCP current is then purely reactive. In SLG faults the positive sequence voltage at the PV terminals stays above 0.9 p.u., the inverters do not enter fault ride-through, and all active modes give the same currents; the recloser phase element then trips more than 1.8 s before the fuse melts, so SLG faults do not constrain the settings. In OM5 the inverters cease for three-phase faults but keep injecting during LL faults, where the terminal voltage is about 0.59 p.u.

**Figure 22:**
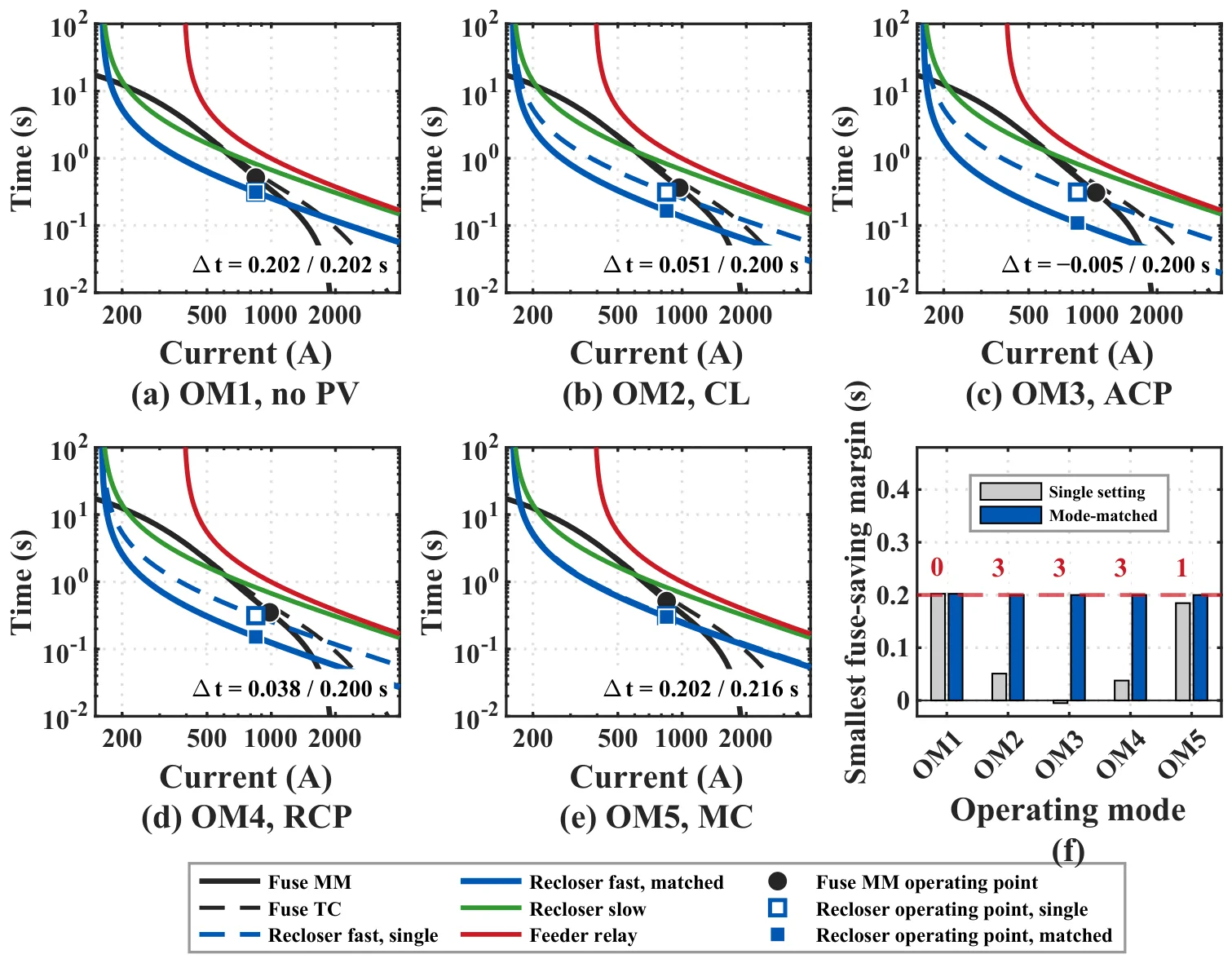
Nediba feeder, three-phase fault at F1. (**a**–**e**) OM1–OM5. (**f**) Smallest fuse-saving margin per mode. Markers: operating points; Δt: fuse-saving margin with the single/mode-matched setting.

With the single setting of the original study, three of the 18 cases violate the fuse-saving margin in each inverter-active mode (three-phase faults at F1 and F2 and the LL fault at F1), and one case does so in OM5 (Table 18). Under ACP the recloser fast curve operates 5 ms after the fuse has started to melt. The mode-matched groups reduce the phase-element fast TDS to 0.0524, 0.0346, 0.0482 and 0.0953 in OM2–OM5, which restores the 0.20 s margin in all 90 cases; the earth element setting is unchanged. The recloser slow curve and the relay are not modified, and the smallest recloser–relay margin for phase faults at F3 is 0.259 s in every mode. Figure 22 shows the curves for a three-phase fault at F1.

The lower fingerprint threshold is $\varphi_{1}=16.8{}^{\circ}$, identical to Case Study I, because it depends only on the current limit and the droop law; the thresholds are therefore network-independent, as the analysis predicts.

## 8. Discussion

### 8.1. Interpretation

The fault current of a GFL inverter is a *controlled* quantity whose angle encodes the active strategy, so the operating mode is observable from a single local measurement. The positive sequence current angle, referenced to the measured voltage, separates CL, ACP and RCP; current magnitude and the sequence ratio resolve MC and asymmetry. Because the thresholds follow from the current limit and the grid code droop law rather than from fitting, $\varphi_{1}$ was identical on both networks. With fixed pickups the coordination sub-problem is linear, so each group is exact, and the online burden reduces to one arctangent, a table lookup and a few comparisons. The sensing layer uses only standard relay measurements. Under class-0.5 noise the group placed in service is coordination-valid in 99.8% of cases, and the residual errors fall where their penalty is smallest.

### 8.2. Comparison with Previous Schemes

Our own earlier scheme [15] uses the same CIGRE benchmark, and its reported fault currents coincide with those used here, so its published TMS vector can be evaluated directly in every mode of this study (Table 19). The fixed setting holds in the utility-only and cessation cases to within 2 ms of the requirement, the rounding of the original study. Once the inverters are active, six pairs violate the margin in each of CL, ACP and RCP, and the worst pair keeps only 0.007 s of the required 0.30 s. Over the five modes, the fixed setting accumulates 24 violations against none for the per-mode groups, which confirms that a single pre-optimized setting cannot maintain selectivity once the inverter mode changes. Its lower total time in these modes results from the violated constraints. The EVPM figures in Table 11 are those reported by its authors on the same benchmark [33]. A numerical comparison with [36] is not meaningful, since that scheme selects settings by demand period on a different network.

### 8.3. Implementation Requirements

The scheme uses only local CT/VT measurements and the multi-setting group feature of commercial relays such as the SEL-751 [41], ABB REF615 [42] and Siemens Reyrolle 7SR21 [43]. These platforms already provide the three functions the scheme needs: local positive- and negative sequence measurement, an inverse-time overcurrent element, and switching between stored setting groups from an internal logic signal. No additional hardware, external signal or firmware feature is required, so the scheme can be commissioned on an installed feeder relay by loading the groups and the threshold table. It stores six setting groups (384 bytes) and a threshold table indexed by 71 voltage and 6 pre-fault current levels (about 3.4 kB), an estimated 4 kB in total, and adds an estimated 10 μs of computation per 4 ms cycle. Both figures are analytical estimates from the data-structure sizes and the operation count of the online cycle, not measurements on a relay platform. The number of setting groups is four in the SEL-751 [41], six in the REF615 (version 5.0 FP1) [42] and eight in the 7SR21 [43]. The REF615 and the 7SR21 therefore hold the six groups of Table 5 directly. On a four-group relay, SG-AMB, which is coordination-valid for both ACP and RCP, replaces SG-ACP and SG-RCP, and the relay holds SG-CL, SG-AMB, SG-MC and SG-AS; the cost is a longer operating time in the ACP and RCP modes, not a loss of coordination. Removing communication also removes the latency, cost and cyber-exposure of alternatives based on supervisory control and data acquisition (SCADA) or IEC 61850 [60] generic object-oriented substation events (GOOSE) (Table 20). Beyond conventional relay setup, commissioning requires one measurement of the inverter reactive gain, the SCR at the PCC for the decision delay of Table 16, and transient-class CTs at relays that carry a coordination pair.

### 8.4. Grid-Forming Inverters

A GFM inverter behaves during a fault as a voltage source $E_{0}∠\theta_{c}$ behind its virtual impedance $Z_{v}$, so its current is set by the network rather than by a priority rule:

(20) $${\underline{I}}^{\mathrm{GFM}}=\frac{E_{0}∠\theta_{c}-{\underline{V}}_{\mathrm{PCC}}}{Z_{v}+Z_{\mathrm{net}}},\;\varphi^{\mathrm{GFM}}=\arg\left(Z_{v}+Z_{\mathrm{net}}\right),$$

referred to the measured PCC voltage. The fingerprint in (20) is still an analytically known angle, but it is fixed by an impedance argument, is largely insensitive to sag depth, and for the inductive $Z_{v}$ of typical designs sits well above the GFL fingerprints. A mixed population is therefore separable by the same decision structure with one additional threshold, provided $Z_{v}$ is known from the interconnection data. What changes is the coordination layer: a grid-forming unit needs its own fault currents and an additional setting group. The classifier structure, the threshold derivation and the LP solution carry over unchanged. Validating this extension requires grid-forming fault studies and is left to future work.

### 8.5. Limitations

- *Simulation-based validation.* The studies use ETAP, MATLAB and an averaged EMT model in Simulink; the scheme has not yet been validated on a real-time simulator or in hardware-in-the-loop, and the classifier uses a statistical instrument transformer noise model rather than recorded relay data.
- *Mixed strategies.* The sensing side of mixed operation is resolved in closed form (Section 4.4), but verifying CTI margins when the two farms operate in different modes requires additional fault cases.
- *Sensing model scope.* CT saturation and PLL re-synchronization are quantified (Section 6.7 and Section 6.6); harmonic distortion and switching-level converter dynamics are not, since the EMT model uses averaged converters.
- *Earth-fault elements.* Asymmetrical faults are assessed for phase elements on an effectively earthed system; resistance-earthed feeders, where earth-fault elements govern SLG faults, are outside the present scope. On the Nediba feeder the inverter share is represented by the current-source model on top of the ETAP network, and only one PV location, the most severe one of [59], is studied.
- *Inverter models and grid codes.* The fingerprints follow IEEE 1547 [12]/2800 [13] and VDE-AR-N 4110 [14]; other droop definitions would shift the thresholds, although the derivation and the classifier structure are unchanged.

## 9. Conclusions

This paper proposed a communication-free adaptive protection scheme for PV-integrated medium-voltage distribution networks in which the active GFL inverter mode is sensed locally from the angle, magnitude and sequence content of the fault current, and a setting group prepared offline for that mode is activated. With fixed pickup currents the coordination sub-problem is a linear program, so every group is computed at its exact optimum; the originally reported metaheuristic vectors were 3–9% above it, and five metaheuristics differed by less than 0.07%. The ACP/RCP ambiguity imposed by the grid code droop law is declared explicitly and served by a group feasible for both modes. Under class-0.5 instrument transformer noise, the group in service is coordination-valid in 99.8% of cases and the expected residual miscoordination is $3.0\times 10^{-5}$ s, 0.01% of the 0.30 s margin, and it does not exceed $7\times 10^{-5}$ s at 2.5 times the class-0.5 noise level. An averaged EMT model confirmed the fingerprints within a few degrees for moderate sags and showed that the PLL settling time, 34–54 ms for a 50 Hz PLL, determines the first-decision delay.

On the CIGRE MV benchmark the scheme kept the required margin in every operating mode for three-phase, LL and SLG faults, and on the Nediba 11 kV feeder model for the same fault types at three fault points. Single-setting coordination produced violations in both cases, including 24 violations of the earlier fixed-setting scheme [15]. The end-to-end test showed a 4.1 ms switching latency with at most 4.4% of the margin affected. The CT saturation and PLL analyses lead to two requirements: transient-class CTs at coordinating relays and an SCR-dependent first-decision delay. Hardware-in-the-loop validation, mixed-strategy coordination and grid-forming units will be addressed in future work.

## Figures and Tables

**Table 1:** Comparison of DOCR coordination studies (2010–2026).

Ref.	Year	Network	Method	Multi- Strat.	V- Adapt.	MC Aware	Asym. Fault	Comm.- Free	GFL Model	Bench.
[23]	2010	IEEE 8-bus	Diff. evol.	✗	✗	✗	✗	✓	✗	Classic
[29]	2011	Distribution	V-adapt. OCR	✗	Partial	✗	✗	✓	✗	Custom
[37]	2016	Nediba 11 kV	Inverter ctrl.	✗	Partial	✗	✓	✓	✓	Egyptian
[20]	2015	IEEE 30-bus	BBO	✗	✗	✗	✗	✓	✗	Classic
[18]	2017	138 kV meshed	Hybrid GA	✗	✗	✗	✗	✓	✗	Real system
[19]	2021	IEEE test MG	Improved PSO	✗	✗	✗	✗	✓	✗	Standard
[30]	2022	Islanded MG	N-1 optim.	✗	✗	✗	✗	✓	✗	IEC MG
[38]	2026	IEEE 3/8/15-bus	Multi-metaheuristic	✗	✗	✗	✗	✓	✗	IEEE
[39]	2024	IEEE bench.	Coati optim.	✗	✗	✗	✗	✓	✗	IEEE
[32]	2024	IEEE-39+8	Dual char.+FCL	✗	✗	✗	✗	✓	Partial	IEEE
[33]	2024	CIGRE+11 kV	EVPM	✗	Partial	Partial	✓	✓	✓	CIGRE
[34]	2022	Microgrid	ANN fault loc.	✗	✗	✗	✗	✗	✗	MG
[35]	2025	AC microgrid	Distributed ML	✗	✗	✗	✗	✗	✗	AC MG
[36]	2024	IIDG	Setting groups	✓	✗	✗	✗	✓	✗	IIDG
[24]	2024	Multi-loop	War optim.	✗	✗	✗	✗	✓	✗	IEEE
[22]	2026	IEC microgrid	GA, GWO	✗	✗	✗	✗	✓	✗	IEC MG
[15]	2025	CIGRE 14-bus	Adaptive (MC)	✗	Partial	✓	Partial	✓	✓	CIGRE
[40]	2025	AC microgrid	Clustering+HIL	Cluster	✗	✗	✗	✓	✓	AC MG
[25]	2025	Complex nets	Walrus optim.	✗	✗	✗	✗	✓	✗	IEEE
**Proposed**	**2026**	**CIGRE+Nediba**	**Fingerprint+LP**	✓	✓	✓	✓	✓	✓	**CIGRE+Nediba**

✓ = addressed; ✗ = not addressed; Partial = partially addressed. Multi-strat. = multi-strategy aware; V-adapt. = voltage-adaptive; MC aware = accounts for momentary cessation; Asym. fault = handles asymmetrical faults; Comm.-free = requires no communication infrastructure; GFL model = explicit grid-following inverter model used; Bench. = benchmark network type; MG = microgrid; LP = linear program; HIL = hardware-in-the-loop testing. Bold denotes the proposed scheme.

**Table 2:** Parameters of the GFL inverter units.

Parameter	Value
AC rating/voltage	1.35 MVA/2 kV
Rated current $I_{r}$	389.7 A
Maximum current $I_{\max}$	$1.20\;I_{r}$
Reactive gain $k_{q}$	2 p.u./p.u.
Deadband $V_{db}$	0.10 p.u.
MC threshold $V_{MC}$	0.50 p.u.
Pre-fault dispatch	$i_{d,0}=I_{r}$, $i_{q,0}=0$

**Table 3:** CT ratios and pickup currents of the relays.

Relay	CT Ratio	$I_{p}$ (A)
OCR_1_–OCR_3_	200/1	50
OCR_4_	200/1	100
OCR_5_, OCR_6_	200/1	150
OCR_7_–OCR_12_	200/1	50
DOCR_1_–DOCR_4_	300/1	100

**Table 4:** Voltage-adaptive fingerprint thresholds ($k_{q}=2$, γ=1).

$|V_{\mathrm{PCC}}|$ (p.u.)	0.90	0.80	0.70	0.60	0.50	0.40	0.30
$\varphi^{\mathrm{ACP}}$ (^∘^)	33.6	33.6	33.6	33.6	33.6	33.6	33.6
$\varphi^{\mathrm{RCP}}$ (^∘^)	9.6	19.5	30.0	41.8	56.4	90.0	90.0
$\varphi_{1}$ (^∘^)	4.8	9.7	15.0	16.8	16.8	16.8	16.8
$\varphi_{2}$ (^∘^)	61.8	61.8	61.8	65.9	73.2	90.0	90.0

**Table 5:** Setting groups and activation triggers.

Group	Trigger	Condition
SG-CL	$\hat{S}=\mathrm{CL}$	Unity power factor
SG-ACP	$\hat{S}=\mathrm{ACP}$	Active current priority
SG-RCP	$\hat{S}=\mathrm{RCP}$	Reactive current priority
SG-AMB	$|\varphi^{\mathrm{ACP}}-\varphi^{\mathrm{RCP}}|\;\leq 2.33\sigma$	ACP/RCP ambiguity band
SG-MC	$|V_{\mathrm{PCC}}|\;<V_{MC}$, $I_{\mathrm{IBR}}<\varepsilon_{I}I_{r}$	Inverter silent
SG-AS	$|I_{2}\left|/\right|I_{1}|\;>0.20$	Asymmetrical fault

**Table 6:** LP-optimal TMS values of the setting groups.

Relay	OM1	OM2 (CL)	OM3 (ACP)	OM4 (RCP)	OM5 (MC+AS)
OCR_1_	0.0500	0.0500	0.0500	0.0500	0.0500
OCR_2_	0.1918	0.2044	0.2026	0.1945	0.1925
OCR_3_	0.3366	0.3707	0.3657	0.3456	0.3351
OCR_4_	0.3787	0.4356	0.4276	0.3958	0.3786
OCR_5_	0.4234	0.4202	0.4208	0.4229	0.4245
OCR_6_	0.5542	0.5517	0.5523	0.5544	0.5560
OCR_7_	0.0500	0.0500	0.0500	0.0500	0.0500
OCR_8_	0.1967	0.2189	0.2159	0.2044	0.1975
OCR_9_	0.3459	0.3417	0.3421	0.3455	0.3475
OCR_10_	0.0500	0.0500	0.0500	0.0500	0.0500
OCR_11_	0.0500	0.0500	0.0500	0.0500	0.0500
OCR_12_	0.2021	0.2237	0.2242	0.2266	0.2273
DOCR_1_	—	0.2231	0.2188	0.2113	—
DOCR_2_	—	0.1337	0.1316	0.1279	—
DOCR_3_	—	0.0500	0.0500	0.0500	—
DOCR_4_	—	0.0500	0.0500	0.0500	—

—: relay not in service (no PV infeed).

**Table 7:** Fault currents (A) and CTI (s) with the matched setting groups.

Fault	Primary/Backup	OM1		OM2 (CL)		OM3 (ACP)		OM4 (RCP)		OM5 (MC+AS)	
		$I_{f}$	CTI	$I_{f}$	CTI	$I_{f}$	CTI	$I_{f}$	CTI	$I_{f}$	CTI
F1	OCR_1_/OCR_2_	1230	0.300	1621	0.300	1559	0.300	1305	0.300	1251	0.300
F2	OCR_2_/OCR_3_	1316	0.300	2098	0.300	1956	0.300	1511	0.300	1251	0.300
F3	OCR_3_/OCR_4_	1355	0.300	2142	0.300	2014	0.300	1576	0.300	1378	0.300
F4	OCR_4_/OCR_5_	1541	0.300	2442/1568	0.300	2295/1568	0.300	1795/1568	0.300	1568	0.300
F5	OCR_5_/OCR_6_	2901	0.300	2949	0.300	2949	0.300	2949	0.300	2949	0.300
F7	OCR_7_/OCR_8_	1371	0.300	2221	0.300	2082	0.300	1621	0.300	1395	0.300
F8	OCR_8_/OCR_9_	1448	0.300	2344/1474	0.300	2198/1470	0.300	1712/1474	0.300	1474	0.300
F9	OCR_9_/OCR_5_	1541	0.561	1568	0.554	1568/1554	0.559	1568	0.554	1568	0.555
F10	OCR_10_/OCR_4_	1335	0.894	2142	0.875	2014	0.876	1576	0.879	1378	0.882
F11	OCR_11_/OCR_12_	1541	0.300	3575/2663	0.300	3422/2663	0.300	2817/2663	0.300	2666/2663	0.300
F13	DOCR_3_/DOCR_2_	—	—	680	0.300	648	0.300	596	0.300	—	—
F14	DOCR_2_/DOCR_1_	—	—	771	0.300	734	0.300	675	0.300	—	—

—: relay not in service (no PV infeed) under that mode. Backup current given after the slash where it differs. F6, F12, F15 and F16 have no backup pair.

**Table 8:** Summary of the LP setting groups.

Mode	*K*	$F^{⋆}$ (s)	Min CTI (s)	Max CTI (s)	Mean CTI (s)	Gap (%) ^1^
OM1 (baseline)	10	12.83	0.300	0.894	0.385	8.98
OM2 (CL)	12	14.33	0.300	0.875	0.369	4.84
OM3 (ACP)	12	14.39	0.300	0.876	0.370	2.97
OM4 (RCP)	12	14.56	0.300	0.879	0.369	4.64
OM5 (MC+AS)	10	12.77	0.300	0.882	0.384	3.01

*K*: number of relays active in that mode; $F^{⋆}$: optimal value of the total operating time in (14). ^1^ Gap of the original metaheuristic solution to the LP optimum.

**Table 9:** Deviation from the LP optimum (%), 30 runs in each of five modes.

Solver	Best	Mean	Std	Worst
RIME	0.0384	0.0613	0.0055	0.0640
GA	0.0606	0.0627	0.0014	0.0640
PSO	$1.1\times 10^{-6}$	0.0026	0.0041	0.0204
WOA	0.0099	0.0208	0.0067	0.0395
GWO	0.0589	0.0615	0.0016	0.0645

**Table 10:** Miscoordination index $M(S_{0}\to S^{\prime })$ ($10^{-2}$ s) for a single-setting scheme.

Group $S_{0}$/Mode $S^{\prime }$	CL	ACP	RCP	MC
CL	0	0.22	1.18	2.14
ACP	0.32	0	0.95	1.85
RCP	1.31	1.01	0	0.66
MC	6.39	6.22	5.47	0
Mean off-diagonal	2.31			

**Table 11:** Comparison with benchmark schemes.

Criterion	Proposed	Single Setting	EVPM [33]
Violations	0	14	3
Minimum CTI (s)	0.300	N/A	0.214
Mean M ($10^{-2}$ s)	0	2.31	0.62
Communication	No	No	No
MC handling	✓	✗	✗
Hardware compatible	✓	✓	✗

✓ = yes; ✗ = no.

**Table 12:** CTI (s) for LL and SLG faults with the three-phase setting groups.

Fault	Primary/Backup	OM1		OM2 (CL)		OM3 (ACP)		OM4 (RCP)		OM5 (MC)	
		LL	SLG	LL	SLG	LL	SLG	LL	SLG	LL	SLG
F1	OCR_1_/OCR_2_	0.315	0.328	0.310	0.319	0.311	0.320	0.314	0.326	0.314	0.328
F2	OCR_2_/OCR_3_	0.314	0.327	0.307	0.313	0.308	0.314	0.311	0.321	0.314	0.328
F3	OCR_3_/OCR_4_	0.327	0.352	0.313	0.324	0.314	0.327	0.321	0.340	0.327	0.351
F4	OCR_4_/OCR_5_	0.330	0.357	0.354	0.404	0.352	0.399	0.339	0.375	0.330	0.356
F5	OCR_5_/OCR_6_	0.316	0.323	0.316	0.323	0.316	0.323	0.316	0.323	0.316	0.323
F7	OCR_7_/OCR_8_	0.314	0.327	0.307	0.313	0.308	0.315	0.311	0.321	0.314	0.326
F8	OCR_8_/OCR_9_	0.314	0.326	0.322	0.343	0.321	0.341	0.317	0.333	0.314	0.326
F9	OCR_9_/OCR_5_	0.613	0.660	0.605	0.651	0.612	0.660	0.606	0.652	0.606	0.652
F10	OCR_10_/OCR_4_	0.949	0.999	0.903	0.926	0.906	0.932	0.923	0.961	0.936	0.985
F11	OCR_11_/OCR_12_	0.314	0.337	0.313	0.334	0.313	0.334	0.312	0.332	0.312	0.331
F13	DOCR_3_/DOCR_2_	–	–	0.300	0.300	0.300	0.300	0.300	0.300	–	–
F14	DOCR_2_/DOCR_1_	–	–	0.300	0.300	0.300	0.300	0.300	0.300	–	–
Min. $I_{f}/I_{p}$		8.9	8.0	6.8	6.8	6.5	6.5	6.0	6.0	9.1	8.1
Σt (s)		13.53	14.12	14.86	15.27	14.94	15.37	15.19	15.69	13.46	14.03
Violations		0	0	0	0	0	0	0	0	0	0

–: relay not in service (no PV infeed) under that mode.

**Table 13:** Decision distribution (%) of the four-output classifier for σ=0.8°.

Active Mode	CL	ACP	RCP	AMB
CL	100.00	0.00	0.00	0.00
ACP	0.00	94.30	0.29	5.41
RCP	0.00	0.31	94.27	5.42
Coordination-valid group	99.80			

**Table 14:** Effect of the angular noise level.

σ (deg)	AMB Band (p.u.)	Valid Group (%)	E[M], 3-Output (s)	E[M], 4-Output (s)
0.4	0.0162	99.91	$3.4\times 10^{-4}$	$1.3\times 10^{-5}$
0.6	0.0244	99.86	$3.6\times 10^{-4}$	$2.1\times 10^{-5}$
0.8	0.0325	99.80	$3.9\times 10^{-4}$	$3.0\times 10^{-5}$
1.0	0.0406	99.75	$4.1\times 10^{-4}$	$3.7\times 10^{-5}$
1.5	0.0609	99.65	$4.9\times 10^{-4}$	$5.2\times 10^{-5}$
2.0	0.0812	99.52	$5.8\times 10^{-4}$	$7.0\times 10^{-5}$

**Table 15:** Threshold sensitivity to the reactive gain $k_{q}$ at $|V_{\mathrm{PCC}}|=0.60$ p.u.

$k_{q}$ (Deviation)	1.6 (−20%)	1.8 (−10%)	2.0 (Nominal)	2.2 (+10%)	2.4 (+20%)
$\varphi^{\mathrm{RCP}}$ (^∘^)	32.2	36.9	41.8	47.2	53.1
$\varphi_{1}$ (^∘^)	16.1	16.8	16.8	16.8	16.8
$\varphi_{2}$ (^∘^)	61.8	63.5	65.9	68.6	71.6

**Table 16:** PLL settling time (ms), EMT model, with the analytical estimate in parentheses.

SCR	$\Delta \theta_{0}$ (deg)	PLL 20 Hz	PLL 50 Hz	PLL 100 Hz
1.5	25.6	187 (59)	– (24)	185 (12)
2	19.8	112 (53)	54 (21)	– (11)
3	13.5	105 (44)	45 (18)	21 (9)
5	8.2	91 (33)	40 (13)	20 (7)
10	4.1	79 (17)	34 (7)	18 (3)

–: PLL not stable.

**Table 17:** Fuse/recloser currents (A) on the Nediba feeder, solid faults, PV at bus 23.

Mode	F1 (Downstream of Fuse)			F2 (End of Lateral)		
	3-Phase	LL	SLG	3-Phase	LL	SLG
OM1 (no PV)	846/846	733/733	395/395	710/710	615/615	329/329
OM2 (CL)	969/846	839/788	453/401	812/690	704/654	377/331
OM3 (ACP)	1034/846	895/753	453/401	867/679	750/609	377/331
OM4 (RCP)	983/846	895/751	453/401	824/691	747/624	377/331
OM5 (MC)	846/846	839/788	453/401	710/710	704/654	377/331

**Table 18:** Fuse-saving margins on the Nediba feeder, 18 cases per mode.

Mode	Single Setting		Mode-Matched			
	Min Δt (s)	Violations	Fast TDS	Min Δt (s)	Violations	Rec.–Relay (s)
OM1 (no PV)	0.202	0	0.0998	0.202	0	0.259
OM2 (CL)	0.051	3	0.0524	0.200	0	0.259
OM3 (ACP)	−0.005	3	0.0346	0.200	0	0.259
OM4 (RCP)	0.038	3	0.0482	0.200	0	0.259
OM5 (MC)	0.185	1	0.0953	0.200	0	0.259
**Total**		**10**			**0**	

Bold denotes the totals row.

**Table 19:** Scheme of [15] and the proposed groups on identical fault currents.

	Ref. [15], Single Setting			Proposed, per-Mode LP		
Mode	Σt (s)	Violations	Min CTI (s)	Σt (s)	Violations	Min CTI (s)
OM1	13.559	3	0.298	12.834	0	0.300
OM2	13.540	6	0.007	14.333	0	0.300
OM3	13.679	6	0.007	14.390	0	0.300
OM4	14.183	6	0.007	14.560	0	0.300
OM5	13.427	3	0.297	12.766	0	0.300
**Total**		**24**			**0**	

Bold denotes the totals row.

**Table 20:** Comparison with communication-based schemes.

Criterion	Proposed	SCADA [29]	GOOSE [30]
Communication	None	Required	Required
Decision latency (ms)	4–190 ^†^	100–500	4–8
Added infrastructure	None	Central master and links	Station bus, IEC 61850 devices
Cyber-exposure	None	High	Medium
Relay settings	6 stored groups	Recomputed centrally	2–4 stored groups
Relay memory (kB)	≈4	0 (remote)	0.1

^†^ Includes the PLL settling time of Table 16.

## Data Availability

The relay-coordination results are contained in the tables of this article. The classifier evaluation and the linear-programming setting groups are reproducible from the analytical model and parameters reported here; the MATLAB scripts and the CIGRE/Nediba network data (ETAP 21.0.2, Operation Technology, Inc., Irvine, CA, USA; MATLAB/Simulink R2024a, MathWorks, Natick, MA, USA) are available from the corresponding author on request.

## Abbreviations

The following abbreviations are used in this manuscript:

ACP	Active Current Priority
AMB	Ambiguous (ACP/RCP) decision
AS	Asymmetrical (fault)
CL	Current Limiting
CT	Current Transformer
CTI	Coordination Time Interval
DOCR	Directional Overcurrent Relay
DG	Distributed Generation
EMT	Electromagnetic Transient
GFL/GFM	Grid-Following/Grid-Forming
GOOSE	Generic Object-Oriented Substation Event
IBR	Inverter-Based Resource
IEC	International Electrotechnical Commission
IIDG	Inverter-Interfaced Distributed Generation
LL	Line-to-Line
LP	Linear Program
MC	Momentary Cessation
MG	Microgrid
MV	Medium Voltage
OCR	Overcurrent Relay
OM	Operating Mode
PCC	Point of Common Coupling
PLL	Phase-Locked Loop
PV	Photovoltaic
RCP	Reactive Current Priority
SCADA	Supervisory Control and Data Acquisition
SCR	Short-Circuit Ratio
SLG	Single-Line-to-Ground
TDS	Time-Dial Setting
TMS	Time Multiplier Setting
VT	Voltage Transformer
